# Emergent flow theory: autonomic coherence as a gain parameter for the temporal architecture of conscious integration

**DOI:** 10.3389/fnint.2026.1841714

**Published:** 2026-09-18

**Authors:** Alfredo López Parra

**Affiliations:** Independent Researcher, Mexico City, Mexico

**Keywords:** autonomic coherence, consciousness, emergent flow theory, heart rate variability, locus coeruleus, Perturbational Complexity Index, salience network, temporal architecture

## Abstract

Contemporary neuroscientific theories of consciousness—including Global Neuronal Workspace Theory (GNW), Integrated Information Theory (IIT), and the Predictive Processing/Free Energy Principle (PP/FEP)—have not formalized autonomic and neuromodulatory state as explicit dynamic control parameters within models of conscious integration. This gap constitutes the explanatory niche addressed by the Emergent Flow Theory (EFT). EFT proposes that consciousness-supporting cortical integration is organized across three nested temporal regimes: ΔTᵢ (autonomic-interoceptive, ~50–200 ms), ΔTₚ (conscious access, ~200–500 ms), and ΔTᶠ (phenomenological-narrative, ~500 ms–3 s)—understood as functional approximations of dominant processing epochs rather than discrete neural compartments. The core theoretical claim is that autonomic coherence (γ), operationally indexed by heart rate variability (HRV), functions as a system-level gain parameter modulating the probability and quality of transitions between these regimes. This relationship is formalized in a phenomenological dynamic equation for a latent integrated-complexity state Q, constrained observationally by measures such as the Perturbational Complexity Index (PCI): dQ/dt = κ·γ·Σ(n,k) [αnk · g(ΔTnk)] − βQ + ξ(t) [*Master Equation — Integrated Cortical Complexity Q* (*γ denotes the dimensionless, latent effective autonomic gain parameter; γ̂ is its empirical estimator, used for calibration and testing but not written into the equation itself*)]. Five empirical predictions (P1–P5), each with explicit adjudication criteria, are derived from this architecture — with P4 comprising a falsifiable existence test (P4a) followed by conditional model discrimination (P4b) — targeting the HRV–PCI covariation profile, transcutaneous vagus nerve stimulation effects on conscious access markers, stress-induced ΔTᶠ disruption, the functional form of the observable γ̂–PCI relationship as an empirical constraint on the latent γ–Q hypothesis, and salience network causal contributions. EFT is offered as a Tier-2 theoretical framework complementary to GNW, IIT, and PP/FEP, situating autonomic coherence as a proposed physiological precondition within which the integrative capacities described by these frameworks may be realized.

## 1 Introduction

### 1.1 The explanatory gap in consciousness science

The question of how unified subjective experience arises from the coordinated activity of distributed neural populations remains one of the central unresolved problems in neuroscience and philosophy of mind. Framed by Chalmers (1995) as the ‘hard problem,’ the challenge is not merely to identify the neural correlates of consciousness—a program that has yielded substantial empirical progress—but to explain why any physical process gives rise to phenomenal experience at all. Nagel (1974) had earlier noted the seemingly irreducible gap between objective description and subjective character, a gap that continues to resist closure despite advances in neuroimaging, electrophysiology, and computational modeling. The present manuscript does not claim to resolve this problem. Rather, it proposes a formal, falsifiable framework—the Emergent Flow Theory (EFT)—that addresses a more tractable but underexplored dimension: the dynamic physiological conditions under which cortical integration sufficient for conscious access may arise and be sustained.

A foundational conceptual distinction is that between access consciousness and phenomenal consciousness (Block, 1995). Access consciousness refers to the availability of information for report, reasoning, and behavioral control—amenable to empirical investigation. Phenomenal consciousness—the subjective, qualitative character of experience—remains more resistant to mechanistic characterization. EFT engages primarily with the access dimension while acknowledging that the relationship between integrative neural dynamics and phenomenal character remains an open theoretical question. The framework is offered as a model of necessary functional conditions, not as a complete theory of consciousness.

### 1.2 The cortical bias of dominant theories

Contemporary theories of consciousness have generated a rich landscape of empirically grounded proposals. The Global Neuronal Workspace Theory (GNW; Dehaene and Changeux, 2011) proposes that conscious access corresponds to a non-linear ‘ignition’ of fronto-parietal networks enabling widespread broadcast of information. Integrated Information Theory (IIT; Tononi et al., 2016) characterizes consciousness in terms of irreducible integrated causal structure (φ); the Perturbational Complexity Index (Casali et al., 2013) is an empirically validated perturbational measure of cortical complexity motivated by related differentiation-and-integration principles, not a direct experimental measurement of φ itself — a distinction sharpened here to avoid the same PCI ≈ φ conflation that this manuscript’s own Q ≠ PCI distinction (§1.4, §5.1) is designed to guard against. The Predictive Processing framework and Free Energy Principle (PP/FEP; Friston, 2005, 2010; Clark, 2013) propose that the brain operates as a hierarchical inference engine minimizing prediction error, with neuromodulatory systems regulating the precision of ascending signals. Each framework has contributed substantially to our understanding, and EFT does not seek to replace any of them.

A shared feature of these frameworks—despite their considerable conceptual differences—is that they have not formalized autonomic and neuromodulatory state as explicit dynamic control parameters within models of conscious integration. GNW does not formally specify neuromodulatory preconditions for ignition threshold accessibility. IIT does not model how structural complexity fluctuates in real time as a function of physiological state. PP/FEP acknowledges neuromodulatory roles but does not provide a temporal model of how autonomic coherence shapes ascending signal gain across discrete processing windows. This shared gap constitutes the explanatory niche EFT is proposed to occupy.

### 1.3 The autonomic dimension: present but underformalised

The relevance of autonomic function to cognition and consciousness is not entirely novel. Thayer and Lane (2009) demonstrated that heart rate variability (HRV) covaries with executive function and prefrontal engagement, proposing a neurovisceral integration model. Seth (2013) and Barrett and Simmons (2015) extended this within a predictive coding framework, arguing that interoceptive signals shape higher-order representations of bodily and affective state. Critchley and Harrison (2013) identified the insular cortex and anterior cingulate as key nodes integrating visceral signals with cognitive processing. Porges (2011) proposed the Polyvagal Theory, in which the myelinated ventral vagal pathway provides a neurobiological substrate for social engagement and psychological flexibility. Converging evidence from transcutaneous auricular vagus nerve stimulation (taVNS) studies in disorders of consciousness further suggests that vagal activation may promote cortical integration through a sequential pathway: NTS activation → LC-norepinephrine and raphe-serotonin modulation → thalamocortical connectivity restoration (Briand et al., 2020). This clinical evidence provides indirect human support for the ascending gain-modulation role assigned to the NTS–LC axis within EFT’s ΔTᵢ regime.

At the systems neuroscience level, converging computational and neuroimaging evidence has established that ascending neuromodulatory projections—particularly from the locus coeruleus—function as intrinsic gain-control parameters capable of driving network-wide transitions in information-processing dynamics (Li et al., 2019; Shine, 2023), providing independent systems-level corroboration of the general principle that ascending arousal systems regulate whole-brain integrative capacity, consistent with the role EFT assigns to γ.

Recent work on interoceptive processing emphasizes the role of brainstem relay structures. The nucleus tractus solitarius (NTS) and the parabrachial nucleus (PBN)—a pontine hub receiving NTS projections and projecting to the amygdala, hypothalamus, thalamus, and insular cortex—constitute successive nodes through which multimodal bodily state information is integrated and transmitted toward cortical regions (Benarroch, 1993; Saper, 2002). What has remained absent is a formal dynamic model integrating autonomic coherence as a quantitative parameter governing the temporal architecture of conscious integration. EFT addresses this gap by introducing autonomic coherence (γ)—operationalized via a dimensionless, normalized HRV-derived estimator (γ̂)—as a gain parameter in a phenomenological dynamic equation situated within a hierarchical temporal architecture.

### 1.4 The emergent flow theory: scope and general proposal

EFT proposes that consciousness-supporting integration is organized across three nested temporal regimes: ΔTᵢ (autonomic-interoceptive, ~50–200 ms), ΔTₚ (conscious access, ~200–500 ms), and ΔTᶠ (phenomenological-narrative, ~500 ms–3 s). These regimes are not rigid compartments but overlapping dominant processing epochs within a continuous dynamical system—their temporal boundaries are functional approximations useful for theoretical organization and experimental design. The core theoretical claim of EFT is that autonomic coherence (γ) functions as a system-level gain parameter modulating the probability and quality of transitions between these regimes, formalized in a phenomenological dynamic equation for a latent integrated-complexity state (Q).

Several clarifications regarding scope are necessary from the outset. First, a normalized HRV-derived estimator is used as an operational proxy for γ; it is not equated with consciousness, nor proposed to directly cause cortical integration. Second, Q is a continuous latent construct; PCI and related neurophysiological markers (P3b, gamma-band synchrony, theta-gamma PAC) are treated as indirect observational constraints on Q, not as its definition and not as causes of consciousness in isolation. This latent/observable distinction is maintained consistently throughout the manuscript (see §5.1 and Table 1). Third, EFT is explicitly a Tier-2 theoretical framework in its current form: grounded in and consistent with a substantial body of empirical literature, but its specific mechanistic claims require direct experimental validation.

**Table 1:** EFT master equation: notation, dimensional closure, and latent/observable status.

Symbol	Units / Range	Definition	Status
Q	Dimensionless [0,1]	Latent integrated-complexity state. Continuous, theoretical; not identical to any single observable.	Latent construct
PCI	Dimensionless [0,1]	Perturbational Complexity Index. A trial-wise observational constraint on Q, not an estimator (Q̂) of it absent a specified measurement model.	Observable proxy
γ	Dimensionless, theoretical	Latent effective autonomic gain. Continuous, theoretical construct — the exact parallel of Q on the autonomic side. Enters the master equation directly as the theoretical gain term; not identified with any single HRV measurement or estimator.	Latent construct (theoretical term in the equation)
RMSSD	ms	Raw, individual-level heart rate variability measurement. An observational constraint on γ, not an identity (γ ≠ RMSSD) and not used directly in the master equation.	Observable proxy
γ̂	Dimensionless (RMSSD / RMSSD_basal)	Operational estimator of γ: raw RMSSD divided by the individual’s own resting baseline RMSSD. Used to empirically calibrate and test the equation’s γ-dependent predictions (§6) — exactly as PCI operationalises but does not equal Q — and does not itself appear as a literal term in the master equation.	Observable estimator (used for calibration/testing, not written into the equation)
γc	Dimensionless, individual-calibrated	Candidate transition region on the γ̂ scale associated with maximal or most rapid change in integrative capacity. Not asserted as a true dynamical critical point pending adjudication among M0/M1/M2 (§5.4); requires Bayesian hierarchical calibration per individual; no longer described as ‘optimal.’	Model parameter
κ	Dimensionless	Scaling constant (to be empirically calibrated).	Model parameter
αnk	s^−1^	Functional integration rate, node n × regime k.	Model parameter
g(ΔTnk)	Dimensionless [0,1]	Temporal coupling kernel; regime–node compatibility.	Model parameter
β	s^−1^	Dissipation constant.	Model parameter
ξ(t)	s^−1^	Additive stochastic noise N(0,σ^2^). Units follow from Q being dimensionless.	Stochastic term
ΔTᵢ	50–200 ms	Autonomic-interoceptive processing regime.	Temporal regime
ΔTₚ	200–500 ms	Conscious access (ignition) regime.	Temporal regime
ΔTᶠ	500 ms–3 s	Phenomenological-narrative integration regime.	Temporal regime

Rows revised in direct response to Reviewer 1 (γ redefined as a latent construct, exact parallel to Q) and Reviewer 2 (dimensional homogeneity; γc reformulated as a candidate transition region, §5.4).

## 2 Current theories and the missing autonomic bridge

### 2.1 Global neuronal workspace theory

The Global Neuronal Workspace Theory (GNW; Baars, 1988; Dehaene et al., 1998; Dehaene and Changeux, 2011) proposes that conscious access corresponds to the ignition of a distributed network of long-range cortical neurons functioning as a global broadcast system. This ignition event has been associated with late electrophysiological signatures under report-demanding paradigms, including a late P3b component of the event-related potential (Sergent and Dehaene, 2004; Sergent et al., 2021) and broadband gamma-band synchrony across fronto-parietal regions; the status of P3b as a direct signature of conscious processing itself, rather than a marker of report or task demands, remains actively debated, particularly in no-report paradigms, and is treated here as a report-dependent access-related marker rather than an unambiguous consciousness signature. From EFT’s perspective, GNW leaves a specific dimension underspecified: the neuromodulatory and autonomic conditions under which the ignition threshold becomes more or less accessible. EFT proposes that autonomic coherence (γ), operating through the LC-NE gain system, constitutes precisely such a parameter—making the frameworks conceptually complementary rather than competing.

### 2.2 Integrated information theory

Integrated Information Theory (IIT; Tononi, 2004; Tononi et al., 2016; Tononi and Koch, 2015) proposes that consciousness is identical to integrated information quantified as φ. The Perturbational Complexity Index (PCI; Casali et al., 2013) has been proposed as a practical proxy, demonstrating strong discriminative validity across states of consciousness (Rosanova et al., 2012). Within EFT, PCI is treated as an observational constraint on the latent construct Q, though the relationship between Q and φ is treated as an open empirical question. IIT’s primary limitation from EFT’s perspective is that it does not model how φ fluctuates as a function of neuromodulatory state. EFT provides a complementary dynamic account of the physiological conditions allowing a system to approach or recede from its maximum integrative capacity.

### 2.3 Predictive processing and the free energy principle

The PP/FEP framework (Friston, 2005, 2010) proposes that the brain operates by minimizing variational free energy. A central concept directly relevant to EFT is precision weighting: neuromodulators implement precision weighting by modulating the gain of neural responses to prediction errors (Feldman and Friston, 2010; Yu and Dayan, 2005). Within EFT, autonomic coherence (γ) can be interpreted as a physiological variable influencing precision weighting of ascending interoceptive and exteroceptive signals—higher γ associated with more reliable interoceptive predictions and more efficient noradrenergic gain modulation. This represents a specific, testable elaboration of the PP/FEP framework rather than an alternative to it.

### 2.4 Identifying the missing bridge

The theoretical landscape reviewed in Sections 2.1–2.3 reveals a convergent explanatory gap. GNW specifies the cortical architecture of conscious broadcast but not the physiological preconditions modulating ignition threshold accessibility. IIT characterizes structural conditions for high integrated information but does not model the dynamic trajectory of φ as a function of neuromodulatory state. PP/FEP formalizes precision weighting by neuromodulators without providing a temporal model of how autonomic coherence shapes ascending signal gain across nested processing windows. Neurovisceral integration and interoceptive predictive coding approaches (Thayer and Lane, 2009; Critchley and Harrison, 2013) establish the empirical relevance of autonomic regulation to cognition but do not formalize its relationship to the temporal dynamics of conscious access.

Recent network neuroscience evidence further documents this gap’s consequence: ascending neuromodulatory gain modulation has been demonstrated to drive whole-brain transitions from information-storage-dominated to information-transfer-dominated states, with a critical transition at intermediate gain values (Li et al., 2019), and phasic bursts of neuromodulatory tone — indexed by pupil diameter — coincide with abrupt large-scale network reconfigurations during perceptual state transitions (Wainstein et al., 2025). To our knowledge, no existing framework formalizes the autonomic precondition of this gain-modulation axis in terms of conscious access architecture.

EFT addresses this gap by introducing autonomic coherence (γ)—operationally indexed by a normalized HRV-derived estimator—as a quantitative, tonic gain parameter that can be empirically estimated, manipulated via non-invasive neuromodulation, and falsified with currently available psychophysiological and neuroimaging tools.

Critically, this precision-weighting account is convergent with Buzsáki's (2006, 2019) oscillatory syntax framework, which demonstrates that the brain segments continuous time into discrete processing windows through hierarchically nested rhythms. While Buzsáki identifies the neurophysiological architecture of this temporal segmentation—phase-amplitude coupling (PAC) between slow rhythms (theta, ~4–8 Hz) and faster oscillations (gamma, ~30–90 Hz), coordinated by fast-spiking parvalbumin-positive interneurons acting as local gain-control gates—EFT operationalises the autonomic precondition of that architecture. Higher autonomic coherence (γ), indexed by HRV, is proposed to modulate cortical gain through LC–NE influences on excitatory–inhibitory balance and gamma-generating parvalbumin-positive interneuron networks (Buzsáki and Draguhn, 2004)—a relationship classified as Tier-2, requiring direct empirical validation of this specific chain in conscious humans. EFT and the oscillatory syntax framework are thus not competing but complementary: Buzsáki’s model maps the cortical mechanism (Tier 1); EFT maps the autonomic precondition of that mechanism’s operation (Tier 2).

## 3 Core temporal architecture of EFT

A foundational premise of EFT is that conscious integration is not a unitary, instantaneous event but a process distributed across multiple temporal scales, each reflecting distinct dominant computational operations and neurobiological substrates. This premise is empirically grounded in the concept of temporal receptive windows (TRWs)—the timescales over which different cortical regions integrate information—as characterized by Hasson et al. (2008). EFT organizes this temporal hierarchy into three functional regimes whose boundaries will vary with individual differences, task demands, and physiological state. They are best understood as dominant processing epochs within a continuous temporal gradient rather than discrete neural compartments (Figure 1).

**Figure 1:**
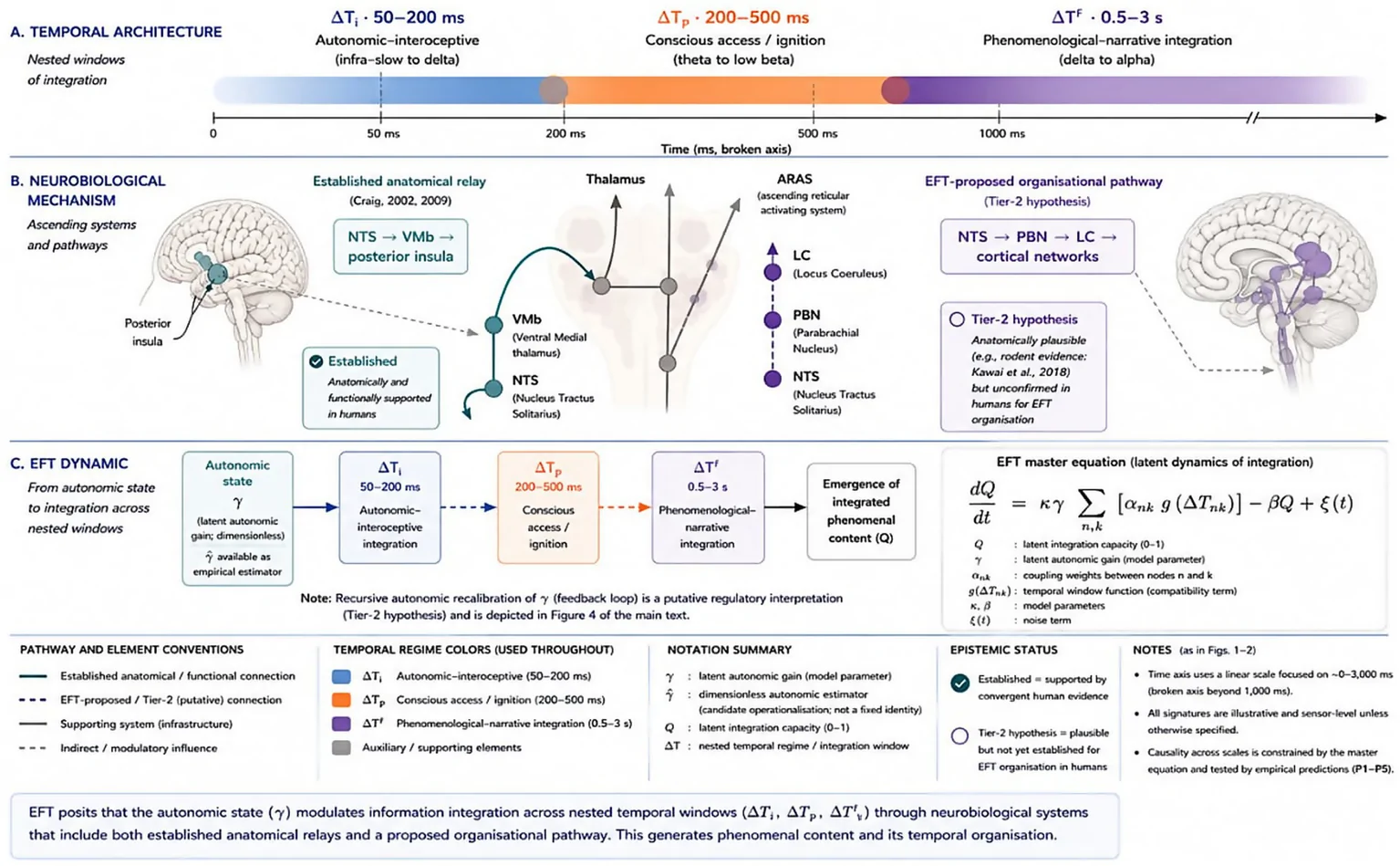
Schematic of EFT’s nested temporal hierarchy. The three regimes (*ΔT*ᵢ, *ΔT*ₚ, *ΔT*ᶠ) are shown as overlapping color-coded bands along a common time axis (~0–3,000 ms), with each regime’s primary anatomical nodes, operational markers, and the master equation positioned centrally. The ascending *ΔT*ᵢ pathway shows two anatomically distinct components: the established NTS → VMb → posterior insular cortex relay (Craig, 2002, 2009) and EFT’s proposed NTS–PBN–LC noradrenergic axis, a Tier-2 hypothesis grounded in rodent tract-tracing evidence (Kawai, 2018). The ARAS is indicated connecting brainstem to thalamus. The Tier-2 designation applies specifically to EFT’s proposed organizational pathway shown in this figure, not to the established NTS → VMb → posterior insula relay, which is independently confirmed and shown as a separate route; the HRV trace is labeled *γ̂*, consistent with Table 1.

### 3.1 ΔTᵢ: the autonomic-interoceptive regime (~50–200 ms)

The first temporal regime is characterized by processing of ascending interoceptive and proprioceptive signals in the sub- and pre-conscious domain. Its functional onset coincides with the arrival of visceral afference at brainstem relay nuclei and its progressive integration toward subcortical and early cortical targets. No phenomenal content is attributed to this regime in itself; it represents a preparatory or permissive phase in which the organism’s baseline regulatory state is established. The dominant process is autonomic integration: the synthesis of cardiovascular, respiratory, and gastrointestinal afferent signals into a coherent representation of bodily state that shapes the gain conditions for subsequent cortical processing.

In non-human primates and humans, Craig (2002, 2009) described a direct pathway from NTS to ventromedial thalamic nuclei (VMb) projecting to posterior insular cortex, providing an established thalamocortical relay for interoceptive afference. This established VMb–insular route is anatomically and functionally distinct from the NTS–PBN–LC noradrenergic axis that EFT proposes as a separate, complementary gain-setting pathway (see §4.1 for the full clarification of what Craig’s work does and does not evidence). This regime corresponds functionally to what Damasio and Carvalho (2013) term ‘primordial feelings’—interoceptive representations of the body’s metabolic state that constitute the neurobiological precursors of the proto-self—without attributing phenomenal character to ΔTᵢ in itself.

Notably, early subcortical relays may respond within ~15–50 ms depending on pathway-specific conduction properties, including degree of myelination and conduction distance; the range of 50–200 ms represents the most conservative empirical estimate for the core integrative window of this regime. A candidate transitional sub-window within this range is discussed in §S1.3 of the Supplementary material.

In its current formulation, EFT explicitly formalizes the autonomic–interoceptive pathway as the primary substrate of ΔTᵢ; the integration of exteroceptive sensory streams—including visual, auditory, and somatosensory inputs—within the same temporal regime constitutes a theoretical extension to be developed in future empirical and modeling work (see §S1.2 of the Supplementary material for a comparative temporal analysis of the visual pathway).

The temporal segmentation underlying ΔTᵢ is consistent with Buzsáki's (2006) oscillatory syntax framework, which demonstrates that the brain organizes information processing into nested temporal windows through phase-amplitude coupling (PAC) between hierarchically arranged oscillatory rhythms. In this framework, slower oscillatory cycles provide the temporal scaffolding within which faster gamma-band activity encodes local content—analogous to the role of ΔTᵢ as the autonomic-interoceptive window that preconditions γ-dependent integration in ΔTₚ. Specifically, EFT proposes—as a Tier-2 mechanistic elaboration—that autonomic coherence (γ) modulates cortical gain through LC–NE influences on excitatory–inhibitory balance and gamma-generating parvalbumin-positive interneuron networks (Buzsáki and Draguhn, 2004; Aston-Jones and Cohen, 2005). Higher HRV-indexed autonomic tone is proposed to be associated with optimal LC phasic activity, which is proposed to support the noradrenergic conditions favoring synchronized gamma-band activity across the ΔTᵢ–ΔTₚ transition. This specific chain—HRV → LC–NE → E/I balance → PV interneuron gamma synchrony → temporal binding—is consistent with converging evidence but has not been validated as a unified circuit in conscious humans and is therefore designated Tier-2 throughout (Figure 2).

**Figure 2:**
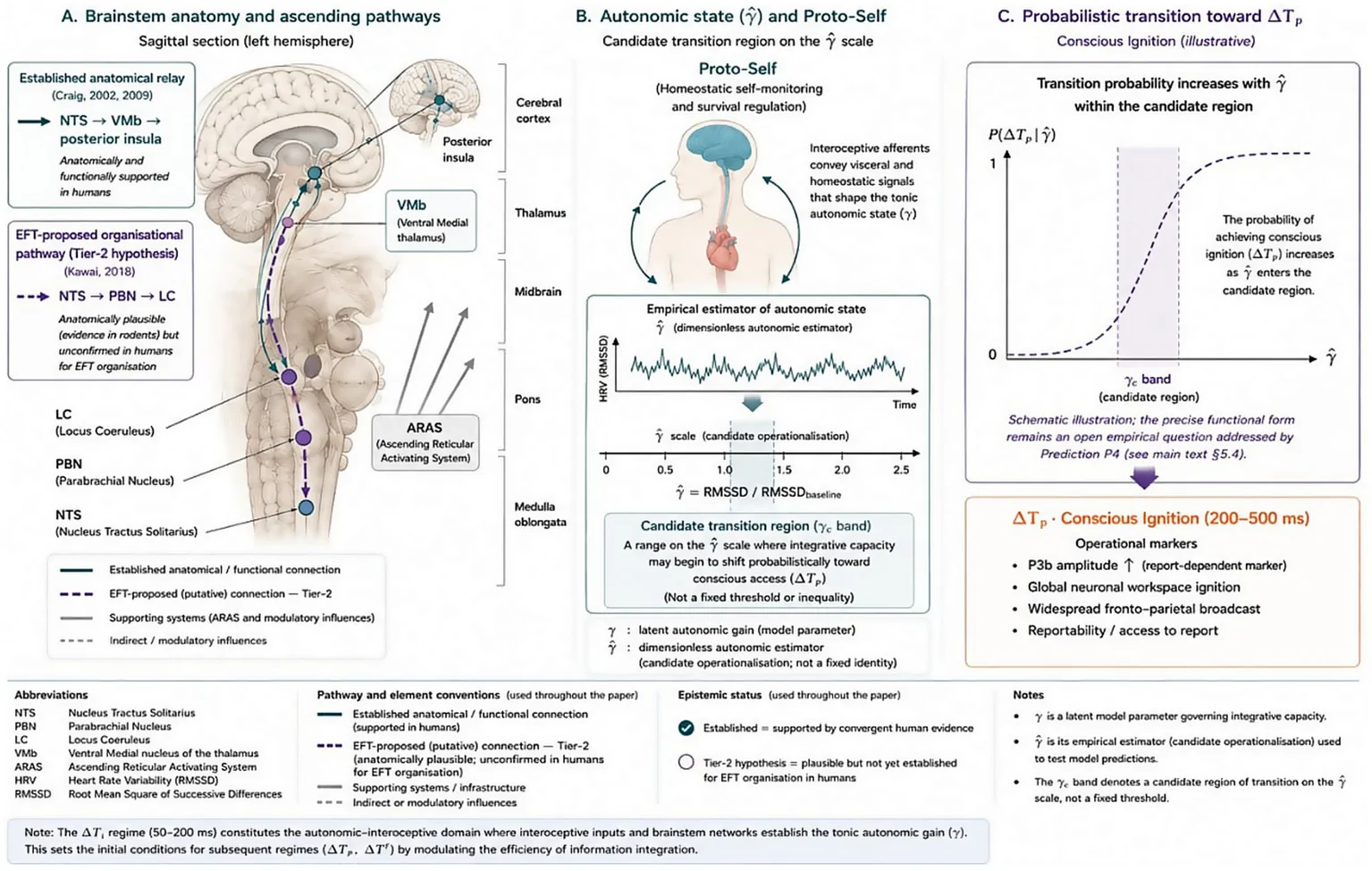
Detailed anatomical diagram of the *ΔTᵢ* regime. Left: Brainstem sagittal section with NTS, PBN, and LC labeled; the ascending NTS → PBN → LC pathway is annotated as an EFT-proposed Tier-2 organizational hypothesis grounded in rodent tract-tracing (Kawai, 2018), anatomically distinct from the established NTS → VMb → posterior insula relay (Craig, 2002, 2009), shown as a separate, independently confirmed route. Center: Autonomic state (*γ̂*) and Proto-Self label, with a candidate observable transition region — not a crisp inequality — indicating where integrative capacity may begin to shift. Right: Probabilistic transition arrow toward *ΔTₚ*/Conscious Ignition. The HRV trace is shown as the empirical *γ̂* estimator, with *γc* marked as a shaded band denoting a candidate transition region on the *γ̂* scale (Table 1), not a fixed threshold line or numeric inequality.

### 3.2 ΔTₚ: the conscious access regime (~200–500 ms)

The second temporal regime corresponds to the window in which, under appropriate conditions, sensory and interoceptive information undergoes a qualitative shift from relatively local, feedforward cortical representations to a globally broadcast, widely distributed neural state—the functional substrate of conscious access as operationally defined. EFT adopts this characterization from GNW (Dehaene and Changeux, 2011) while extending it to include the modulatory role of antecedent autonomic state. The dominant process is global ignition: a non-linear, self-amplifying cascade of activity across fronto-parietal workspace neurons (Figure 3).

**Figure 3:**
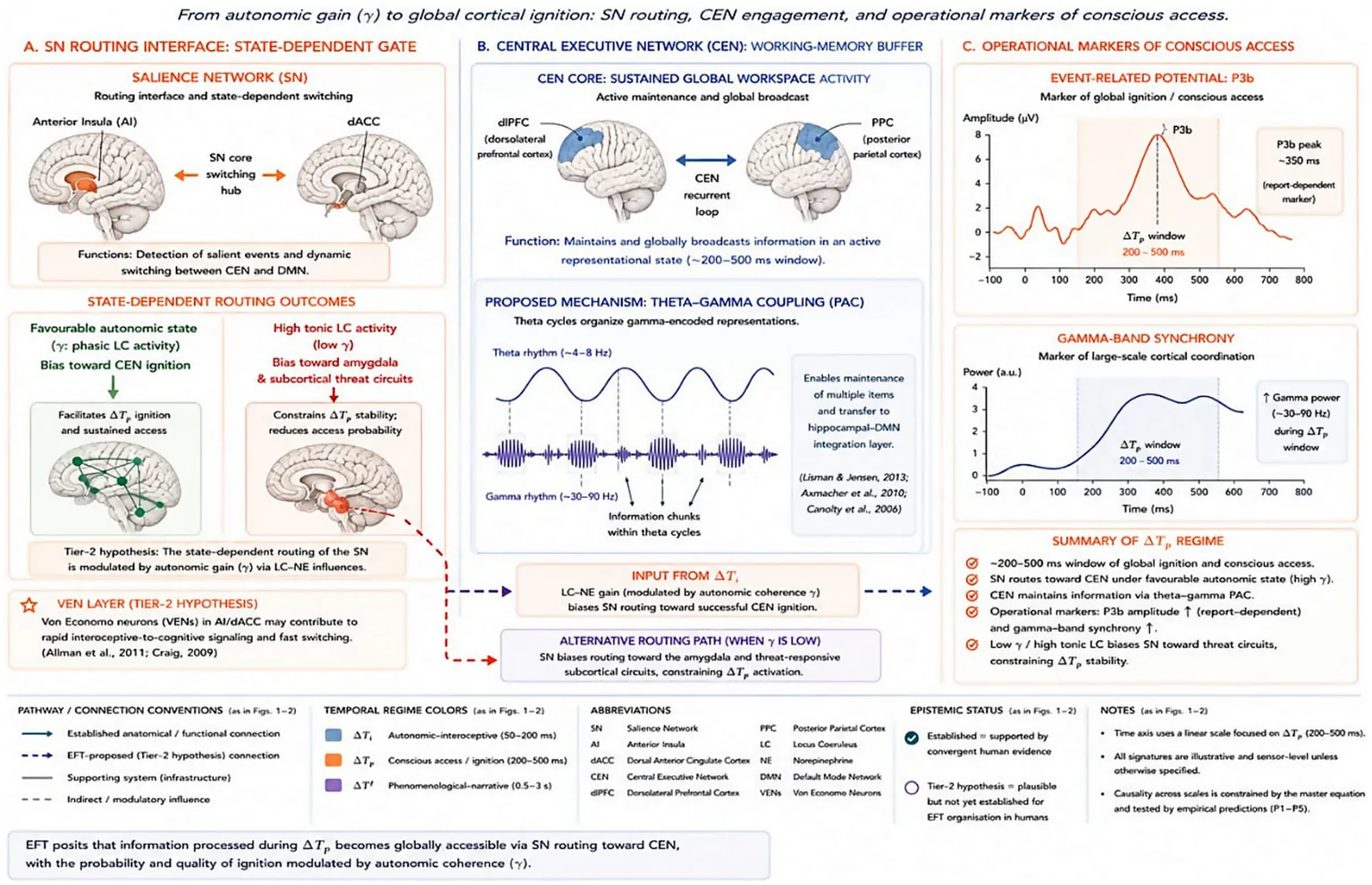
Diagram of the ΔTₚ regime. Left: SN routing interface (anterior insula + dACC) with the VEN layer labeled as a Tier-2 hypothesis. Center: CEN (dlPFC + posterior parietal cortex) as working-memory buffer. Right: Schematic EEG waveform showing P3b peak at ~350 ms with gamma-band synchrony overlay. Arrows indicate LC-NE gain input (from ΔTᵢ) → SN → CEN; an alternative routing path to the amygdala is shown as a dashed line.

### 3.3 ΔTᶠ: the phenomenological-narrative regime (~500 ms–3 s)

Within the EFT architecture, ΔTᶠ begins once information maintained within the CEN working-memory buffer becomes available for hippocampal–cortical integration and autobiographical contextualisation. Its functional onset is contingent on the successful completion of CEN buffering during ΔTₚ and is marked by the transition from fronto-parietal dominance to hippocampal-DMN engagement. ΔTₚ represents the functional moment of global access; ΔTᶠ represents the subsequent period during which that information is integrated into the organism’s ongoing narrative representation—contextualized by episodic memory, situated within a temporal self-model, and structured by the hierarchical linguistic and conceptual frameworks that characterize narrative comprehension (Xu et al., 2005). Damasio (1999) distinguished between a ‘core self’ tied to the immediate present and an ‘autobiographical self’ constituted by the continuity of personal memory. ΔTᶠ concerns the short-timescale neural operations through which currently accessed information may be contextualized within self-related and autobiographical representations; it should not be equated with Damasio’s autobiographical self itself, which — as clarified below — properly refers to a much longer-timescale construct. This relationship is best understood as a functional alignment with the process of contextualisation Damasio describes, not an ontological identification with either level of his self-hierarchy.

It should be noted that in Damasio’s original framework, the autobiographical self extends over timescales of weeks to years, considerably beyond EFT’s temporal architecture. ΔTᶠ (~500 ms–3 s) is temporally more proximate to Damasio’s core self — the moment-to-moment sense of self generated by ongoing body-state representation — than to the full autobiographical self. EFT claims correspondence with the core self temporal scale; the autobiographical self in Damasio’s sense lies beyond EFT’s current temporal scope and is not claimed here.

The neurobiological substrate of ΔTᶠ is anchored in the Default Mode Network (DMN), which supports autobiographical memory retrieval, prospective mental simulation, and theory of mind—functions that share a common neural basis within the medial prefrontal and medial temporal subsystems (Raichle et al., 2001; Buckner et al., 2008; Spreng et al., 2009). Within the DMN, functional fractionation studies reveal distinct subsystems—a medial temporal lobe component (hippocampus, parahippocampal cortex) specialized for memory encoding and retrieval and a medial prefrontal component (vmPFC, posterior cingulate cortex) specialized for self-referential processing (Andrews-Hanna et al., 2010)—whose coordinated engagement constitutes the neurobiological substrate of ΔTᶠ integration. The temporal receptive windows of DMN nodes are among the longest in the cortex (Hasson et al., 2008), making the DMN structurally suited to sustain the extended integration characteristic of this regime (Figure 4).

**Figure 4:**
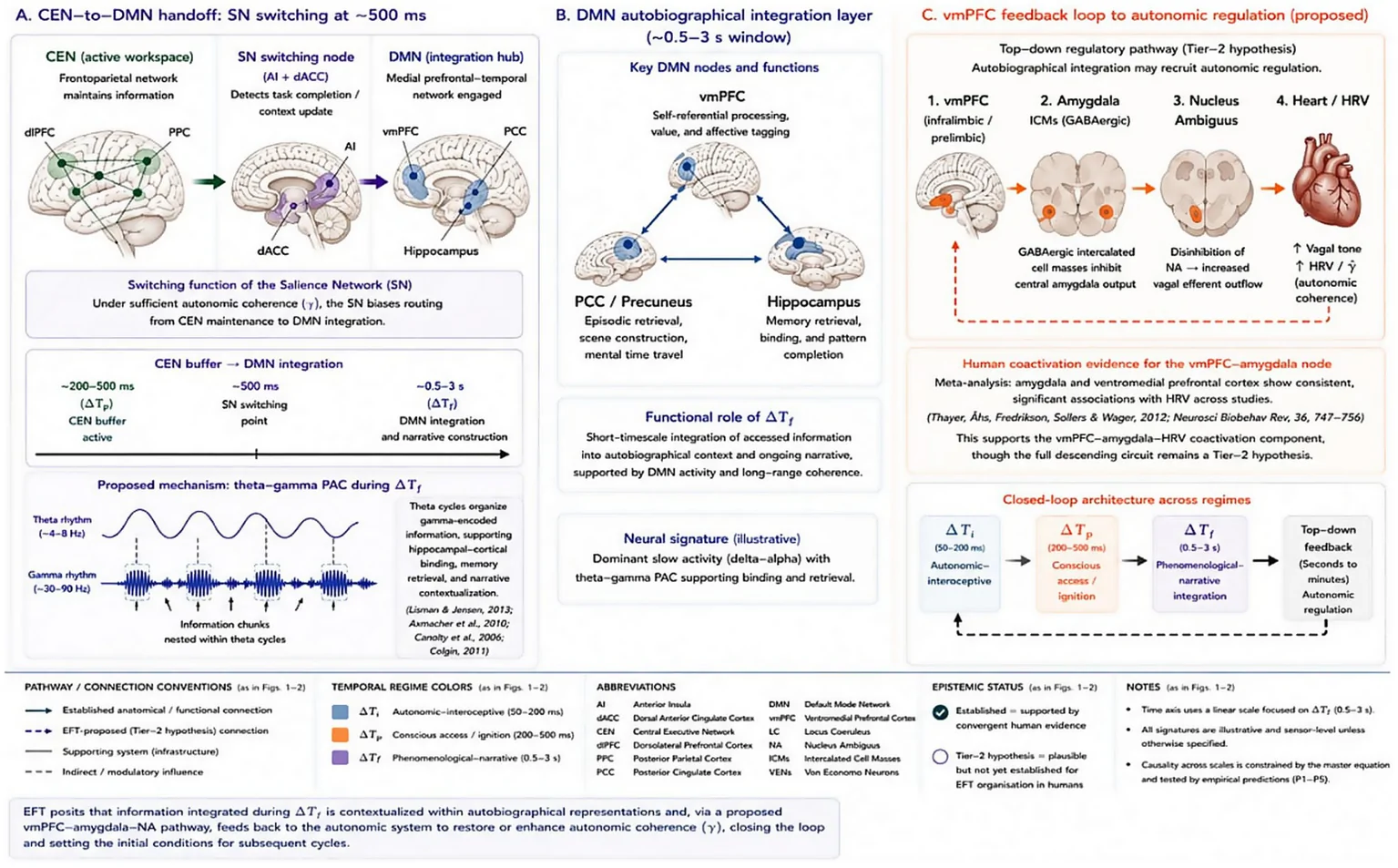
Diagram of the *ΔTᶠ* regime and vmPFC feedback loop. Left: CEN-to-DMN handoff, mediated by SN switching. Center: DMN nodes (vmPFC, PCC, hippocampus) as the autobiographical integration layer, with a theta-gamma PAC schematic. Right: Descending feedback pathway (vmPFC → amygdala ICMs → nucleus ambiguus → ↑*HRV*/↑*γ̂*), closed-loop arrow returning to *ΔTᵢ*. The vmPFC–amygdala node is annotated with its supporting human coactivation evidence (Thayer et al., 2012; see §4.4). The descending feedback pathway remains a putative closed-loop regulatory interpretation (Tier-2).

### 3.4 The nested architecture: temporal regimes as mutually constraining conditions

The three temporal regimes are not merely successive processing steps but mutually constraining functional conditions. The quality of processing within ΔTᵢ—as indexed by γ—may influence the probability and amplitude of the ΔTₚ ignition transition by modulating ascending neuromodulatory gain. In turn, the richness and stability of ΔTₚ integration may determine the quality of material available for ΔTᶠ autobiographical consolidation. EFT also proposes a top-down feedback component: regulatory processing originating in the vmPFC during ΔTᶠ may modulate autonomic outflow through GABAergic intercalated cells in the amygdala, thereby releasing inhibitory constraint on the nucleus ambiguus and supporting restoration of ventral vagal tone (Quirk and Mueller, 2008; Critchley and Harrison, 2013). This descending pathway is classified as Tier-2, consistent with the emotion regulation literature (Thayer and Lane, 2009; Ochsner and Gross, 2005; Arnsten, 2015) but requiring direct empirical validation within the EFT framework. It does not constitute a demonstrated causal circuit in conscious humans.

## 4 Dynamic routing mechanisms

The temporal architecture described in Section 3 requires neurobiological routing mechanisms that translate the organism’s autonomic state into differential patterns of cortical engagement across regimes. The proposed multi-node routing pathway—NTS-PBN → LC → SN → CEN → DMN → vmPFC feedback—should not be interpreted as a strictly serial chain but as a probabilistic influence network in which multiple nodes interact dynamically. Language is calibrated accordingly throughout: may, is proposed to, increase the probability of, bias toward, and facilitate mark the inferential status of each claim.

### 4.1 The NTS-PBN axis as interoceptive afference relay

The NTS of the dorsal medulla constitutes the brain’s primary first-order relay for visceral afferent signals conveyed by the vagus nerve (Benarroch, 1993; Saper, 2002). The NTS is a recipient of ascending vagal afference; cardiovagal efferent outflow arises prominently from the nucleus ambiguus, while the dorsal motor nucleus of the vagus (DMNX) contributes importantly to other parasympathetic visceral functions (Porges, 2011).

Rodent anterograde tract-tracing provides the anatomical basis for the ascending routes discussed in this section. Kawai (2018) used biotin dextran amine (BDA) injections into the caudal NTS in rat and reported anterogradely labeled axon terminals in the lateral parabrachial nucleus and in related global-projection targets; this evidence is rodent-specific, and documents anatomically distinct ascending routes rather than establishing a single confirmed serial NTS → PBN → LC chain in any species. The PBN provides a multimodal interoceptive integration hub that may elaborate and contextualize NTS afference before influencing LC gain-setting during ΔTᵢ, but the specific serial chain proposed here — as distinct from parallel NTS → PBN and NTS → LC projections independently documented by Kawai (2018) — is EFT’s own organizational hypothesis, not a directly demonstrated single circuit, and is labeled Tier-2 accordingly.

A separate and independently established ascending route is documented in primates and humans: Craig (2002, 2009) described a direct projection from NTS to ventromedial thalamic nuclei (VMb), relaying to posterior insular cortex, providing the anatomical basis for Craig’s account of interoceptive representation and anterior-insular awareness. This VMb–insular route is anatomically and evidentially distinct from the NTS–PBN–LC route discussed above; the two should not be conflated as a single pathway. Three evidentiary layers accordingly remain distinct throughout this manuscript: (i) established primate/human evidence — the NTS–VMb–posterior insula relay (Craig, 2002, 2009); (ii) supported rodent components — the individual NTS → PBN and NTS/PBN region→LC links (Kawai, 2018); and (iii) EFT’s own synthesis — the proposal that these rodent-documented components compose a functional NTS–PBN–LC gain-setting axis operating in humans, an original theoretical construct of this manuscript rather than an inherited finding from any single cited source.

Because the NTS → PBN → LC route and the NTS → VMb → insula route are not confirmed to be the same anatomical chain, any experimental design that infers ‘target engagement’ at the NTS from downstream cortical or thalamic readouts cannot assume the two routes are interchangeable. A tVNS or vagal-activation protocol intended to test EFT’s proposed gain-setting axis must therefore be designed to dissociate — insofar as available methods allow — PBN/LC-mediated noradrenergic effects from VMb/insula-mediated thalamocortical effects, rather than treating any downstream interoceptive signature as confirmation of the specific route EFT proposes. At present no validated biomarker achieves this dissociation in humans (Yap et al., 2020; see §7.1); this is an explicit design constraint on future tVNS studies within the EFT program.

Indirect functional evidence in humans is provided by taVNS studies in disorders of consciousness demonstrating thalamocortical connectivity restoration following vagal activation (Briand et al., 2020); this evidence is consistent with EFT’s general claim that vagal afference promotes cortical integration, but does not by itself discriminate between the VMb–insular route and the PBN–LC route as the operative mechanism.

### 4.2 Locus coeruleus–norepinephrine gain modulation

The LC is the brain’s principal noradrenergic nucleus, projecting widely throughout the neuroaxis (Foote et al., 1983). Aston-Jones and Cohen (2005) proposed a model in which the LC operates in phasic (stimulus-locked bursts, optimal performance) and tonic-high (sustained elevated NE, degraded signal-to-noise) modes. Intermediate or functionally favorable autonomic coherence (γ) is proposed to be associated with LC phasic activity; reduced γ may be associated with tonic-high activity degrading fronto-parietal signal discrimination and raising the effective ignition threshold. NE facilitates cortical responsiveness and modulates prefrontal network function in a state-dependent manner (Arnsten et al., 2012; Aston-Jones and Cohen, 2005). Sara and Bouret (2012) provide a complementary account in which LC activation mediates cognitive reorientation through arousal-dependent gain modulation across multiple cortical targets.

At the whole-brain network level, Li et al. (2019) demonstrated computationally and empirically that ascending neuromodulatory gain modulation drives a critical transition in information processing dynamics—from information-storage-dominated (subcritical) to information-transfer-dominated (supercritical) states—with a transition occurring at intermediate gain values, providing a systems-level precedent for the candidate transition region EFT denotes γc, without itself establishing γc as a validated critical point (§5.4). Operating near this critical regime provides computational advantages—flexibility in information processing achievable through only subtle changes in gain control—that the γ · αnk term formalizes mechanistically. Shine (2023) further established that diffuse neuromodulatory projections enforce low-dimensional neural dynamics promoting system-wide coherence while maintaining susceptibility to contextually-driven state transitions—a property directly instantiated in EFT’s model as the γ-dependent modulation of the ΔTᵢ–ΔTₚ transition probability. This chain of evidence is systems-level and correlational; the specific claim that cardiac-vagal HRV (rather than neural gain measured directly) is the operative upstream driver remains a Tier-2 EFT-specific extension requiring direct empirical validation.

Dopaminergic (VTA/SN) signaling—implicated in effort-based motivational computation and incentive salience (Salamone and Correa, 2012)—and glucocorticoid signaling—whose chronic dysregulation produces measurable structural and functional changes in the prefrontal–hippocampal–amygdala circuit (Ulrich-Lai and Herman, 2009)—are interpreted as second-order contextual modulators of the primary NTS-LC-SN routing pathway, not primary drivers of the temporal architecture (Joëls and Baram, 2009). This interpretive framing preserves parsimony while acknowledging the documented contributions of multiple neuromodulatory systems to state-dependent cortical regulation.

### 4.3 The salience network as state-dependent routing interface

The Salience Network (Menon and Uddin, 2010; Menon, 2011), anchored by the anterior insula (AI) and dorsal anterior cingulate cortex (dACC), performs two principal functions: detection of salient events and dynamic switching between the CEN and DMN. The right fronto-insular cortex (rFIC) plays a particularly critical causal role in this switching, initiating CEN activation and DMN suppression across multiple task paradigms and stimulus modalities (Sridharan et al., 2008). Under conditions of optimal γ and phasic LC activity, the SN may bias routing toward the CEN, increasing the probability of sustained conscious access and subsequent hippocampal-DMN integration. Under conditions of elevated tonic NE—associated with hyperarousal or acute threat—SN routing may be biased toward amygdala and subcortical threat-responsive circuits (Seeley et al., 2007), constraining the stability and duration of the ΔTₚ access window. This routing bifurcation is a Tier-2 state-dependent tendency rather than a deterministic switch.

Von Economo neurons (VENs), which are disproportionately concentrated in the AI and dACC and morphologically specialized for rapid long-range axonal conduction, have been proposed as candidate cellular substrates for the fast interoceptive-to-cognitive signaling that underlies SN routing (Allman et al., 2011; Craig, 2009). Their functional role within the ΔTᵢ–ΔTₚ transition—potentially mediating the speed and fidelity of autonomic-state signals reaching SN nodes—constitutes a Tier-2 cellular hypothesis for future investigation, consistent with their concentration in precisely those cortical regions EFT assigns to the routing interface. This candidate mechanism (VENs) and the switching mechanism documented by Sridharan et al. (2008) (rFIC causal role in CEN/DMN switching) are two independently sourced claims that jointly motivate the routing interface; Sridharan et al. (2008) does not itself report on VENs, and the two lines of evidence should not be conflated as a single finding.

Recent empirical evidence provides a further, independent anchor for gain-dependent state-routing: Wainstein et al. (2025) and colleagues demonstrated that qualitative perceptual switches co-occur with phasic peaks in pupil diameter—an indirect index of neuromodulatory tone—and that recurrent neural network modeling and fMRI data together support the interpretation that increased gain drives these network reconfigurations. This is offered as a prospective empirical anchor for EFT’s Prediction P2, pending direct HRV-indexed replication, and is classified Tier-2.

### 4.4 CEN, DMN, and the putative closed-loop feedback

The CEN (Miller and Cohen, 2001; Curtis and D'Esposito, 2003) maintains information in an active representational state through sustained recurrent prefrontal-parietal activity. The proposed electrophysiological mechanism is theta-gamma PAC (Lisman and Jensen, 2013; Axmacher et al., 2010; Canolty et al., 2006): each theta cycle organizes gamma-encoded representations, enabling maintenance of multiple items and transfer to the hippocampal system.

Hippocampal–prefrontal oscillatory coupling during the ΔTₚ–ΔTᶠ transition further supports this architecture: Colgin (2011) demonstrated that distinct gamma-frequency sub-bands index local processing versus long-range communication in hippocampal circuits, providing a mechanistic basis for the CEN-to-DMN information transfer proposed in EFT’s ΔTᶠ regime. Prefrontal working memory is highly sensitive to the inverted-U catecholamine relationship: excess NE degrades delay-period firing precision (Arnsten, 2015; Arnsten et al., 2012), truncating the ΔTₚ window. vmPFC projections to GABAergic intercalated cell masses of the amygdala may recruit inhibition of central amygdala output to the DMNX and hypothalamus, reducing sympathetic drive and potentially restoring ventral vagal tone (Quirk and Mueller, 2008; Likhtik et al., 2008).

This putative closed-loop feedback is classified as Tier-2. Its cellular-level components draw on the emotion-regulation literature (Ochsner and Gross, 2005; Arnsten, 2015), and its human systems-level grounding is strengthened here in direct response to peer review: Thayer et al. (2012) — an existing, DOI-verified reference in this manuscript — report a meta-analysis of HRV–fMRI neuroimaging studies identifying the amygdala and ventromedial prefrontal cortex as regions showing consistent, significant associations with HRV across studies, providing direct human coactivation evidence for the vmPFC–amygdala node of this feedback pathway. This finding constrains but does not establish the full descending circuit posited here: it demonstrates vmPFC–amygdala–HRV coactivation, not the specific GABAergic intercalated-cell mechanism or its causal direction, which remain Tier-2 proposals requiring direct empirical validation within the EFT framework.

## 5 Dynamic model of integrated complexity

### 5.1 Overview and the master equation

The core dynamic equation of EFT is (*Master Equation — Integrated Cortical Complexity Q*):


dQ/dt=κ·γ·Σ(n,k)[αnk·g(ΔTnk)]−βQ+ξ(t)

The equation is a phenomenological systems model—a mathematical scaffold organizing the conceptual relationships between EFT’s parameters in a tractable, falsifiable form—analogous in spirit to neural mass models (Deco et al., 2008). It characterizes functional operations at the meso-scale of systems neuroscience and does not constitute a biophysical derivation from molecular mechanisms. Two structural clarifications are introduced here directly in response to peer review, and are maintained in exact parallel with one another. First, Q is defined as a continuous latent state; PCI is a trial-wise observational constraint on Q, not an identity (Q ≠ PCI) and not yet a formal estimator (Q̂) in the absence of a specified measurement model linking the two — and PCI does not itself appear as a term in the equation. Second, and in exact structural parallel, γ is defined as a continuous latent theoretical construct — a dimensionless effective autonomic gain — rather than as raw HRV in milliseconds; this resolves the dimensional inconsistency identified in review at the level of the equation’s own primitive terms, rather than by substituting an empirical proxy into the theoretical core. With γ dimensionless, κ dimensionless, αnk in s^−1^, and g(ΔTnk) dimensionless, the product κ·γ·αnk·g(ΔTnk) carries units of s^−1^, matching both dQ/dt and −βQ (β in s^−1^, Q dimensionless). Table 1 provides the complete dimensional closure for all model terms.

The equation is therefore stated entirely in terms of latent theoretical quantities (Q, γ). Empirical calibration and testing of its γ-dependent predictions (§6) use γ̂ = RMSSD/RMSSD_basal, the operational estimator of γ, exactly as PCI is the operational proxy used to test its Q-dependent predictions — neither observable is substituted into the equation itself. This maintains a single consistent architecture across both latent/observable pairs (Q/PCI and γ/γ̂), avoiding an asymmetry in which one pair’s measurement instrument enters the theoretical core while the other’s does not.

Within each HRV estimation epoch, γ is treated as a quasi-static latent state — approximately constant over the timescale at which γ̂ is estimated — that conditions the faster dynamics of Q, rather than as a quantity recalculated at the timescale of ΔTᵢ or ΔTₚ. This quasi-static treatment is what makes it coherent for a tonic/baseline construct to appear as a multiplicative term within an instantaneous differential equation; see also §7.1 for the corresponding measurement-scale limitation.

A population-level numeric anchor for γc (e.g., a specific RMSSD range in healthy adults) was present in earlier versions of this manuscript, attributed to Thayer et al. (2012). On review, that attribution could not be independently confirmed against the cited meta-analysis with the same confidence as the manuscript’s other verified claims from that source (its confirmed content concerns HRV–fMRI regional associations, not RMSSD population percentiles), and the specific figure has accordingly been removed from Table 1 rather than retained on an unverified citation. γc is calibrated exclusively on the dimensionless γ̂ scale via individual-level Bayesian hierarchical estimation; no population-level millisecond anchor is required by the model as presented, particularly for patient populations with vagal neuropathy or chronic autonomic dysfunction (§7.2), where the mapping between raw RMSSD and γ̂ may deviate substantially from healthy-population norms in any case.

Parameter identifiability. Although the specific numerical values of κ, β, αnk, and g(ΔTnk) remain to be empirically calibrated, the model is not parameter-free. The following boundary conditions are imposed by theoretical necessity: κ > 0; β > 0 and finite; g(ΔTnk) ∈ [0, 1]; and γ ≥ 0. Its empirical estimator is calibrated such that γ̂ = 1 corresponds to an individual’s own resting baseline. Holding all other model terms constant, the first-order driving term κ·γ·Σαnk·g(ΔTnk) increases with γ by construction. This local structural property of the equation should not be interpreted as specifying the organism-level functional relationship at the observable level — between γ̂ and PCI — which depends on the interaction of this driving term with the dissipation term −βQ and remains to be adjudicated empirically among the candidate models described in §5.4, and which in any case constrains rather than directly estimates the latent γ–Q relationship in the absence of a measurement model linking PCI to Q. Conflating the equation’s local structural monotonicity with a claim about the observable-level relationship was an imprecision in earlier versions of this manuscript, corrected here directly in response to peer review.

### 5.2 Gain-modulated integration: the γ · αnk term

The multiplicative structure of the γ · αnk term reflects gain modulation—a canonical computational operation of the central nervous system (Salinas and Thier, 2000). γ scales the effective integration rate αnk at every node-regime pair in proportion to current autonomic coherence; empirically, this scaling relationship is calibrated and tested using the normalized estimator γ̂ (§6). Servan-Schreiber et al. (1990) demonstrated computationally that norepinephrine enhances signal-to-noise ratios through multiplicative gain modulation; Aston-Jones and Cohen (2005) provided the physiological context: phasic LC activity amplifies responses to task-relevant signals in a manner consistent with the γ · αnk term.

The scaling of this gain-modulation architecture from cellular to systems level is empirically documented: Shine et al. (2019) demonstrated that large-scale neural activity across diverse cognitive tasks converges onto a low-dimensional manifold, with the axes of this neurocognitive architecture aligning with regional differences in neuromodulatory receptor density. This finding provides network-level evidence that the gain-modulation mechanism described by Aston-Jones and Cohen (2005) at the cellular level operates coherently at the meso-scale at which Q is operationalized in EFT.

This structure generates a specific prediction: two individuals with identical αnk profiles may show markedly different Q dynamics if their γ values differ (as estimated empirically by γ̂).

An interpretive analogy—offered as a Tier-2 scaffold, not a strict mechanistic claim—draws on NMDA receptor biophysics: NMDA receptors function as molecular AND-gates requiring both presynaptic neurotransmitter release (analogous to αnk) and sufficient postsynaptic depolarisation (analogous to γ) to relieve the Mg^2+^ block and sustain recurrent excitation consistent with ignition (Markram et al., 1997; Larkum, 2013).

### 5.3 Temporal coupling kernel g(ΔTnk)

The temporal coupling kernel g(ΔTnk) specifies the effective contribution of temporal regime k to integration at node n as a function of the temporal window ΔTnk—conceptually the compatibility between a node’s intrinsic TRW and the temporal scale of the processing regime. Three neurobiologically motivated Tier-2 interpretations: (1) the TRW profile of node n for regime k, as empirically characterized by Hasson et al. (2008); (2) the cross-frequency coupling envelope governing effective theta-gamma communication (Canolty and Knight, 2010; Lisman and Jensen, 2013); or (3) phase-dependent communication windows wherein effective synaptic transmission is maximized at specific phase relationships (Fries, 2005). The explicit mathematical form requires empirical constraint.

A fourth interpretation, consistent with Buzsáki's (2006, 2019) oscillatory syntax, proposes that g(ΔTnk) encodes the hierarchical phase-amplitude coupling (PAC) relationship between slow carrier oscillations and fast gamma-band activity: g(ΔTnk) is maximal when the temporal regime k aligns with the intrinsic oscillatory scale at which node n preferentially computes. This interpretation provides a biophysically grounded account of why temporal windows of ~50–200 ms (ΔTᵢ), ~200–500 ms (ΔTₚ), and ~500 ms–3 s (ΔTᶠ) correspond to distinct dominant computational operations: each maps onto a different dominant oscillatory frequency band in Buzsáki’s logarithmic rhythm hierarchy.

A fifth interpretive direction, grounded in computational neuroscience of sequential cognition, proposes that the cascade ΔTᵢ → ΔTₚ → ΔTᶠ is consistent with metastable attractor networks characterized by heteroclinic channels (Rabinovich et al., 2008): each temporal regime constitutes a metastable state connected to the next by a transient heteroclinic trajectory. In this framework, γ modulates the depth and transition speed of each metastable state — higher autonomic coherence (as indexed empirically by γ̂) associated with more stable transient states and more efficient ΔTₚ ignition. This interpretation is Tier-2 and consistent with the broader class of sequential attractor dynamics documented in neural systems (Rabinovich et al., 2014).

### 5.4 Dissipation, noise, and the functional form of the γ–Q relationship

The dissipation term −βQ captures the thermodynamic property that integrated cortical states are metabolically costly and decay toward baseline in the absence of sustained driving input (Attwell and Laughlin, 2001). The stochastic term ξ(t) represents intrinsic neural variability modeled as additive Gaussian noise (Deco et al., 2008).

A substantive revision to the model’s empirical claims is introduced in this section, directly addressing peer review. Earlier versions of this manuscript predicted a purely sigmoidal (monotonically saturating) γ–Q relationship. Reviewer 1 correctly noted that this conflicts both with the reservoir-computing and neural-criticality literature — which characterizes computation near a critical point as a sharply peaked, not monotonic-saturating, function of the control parameter (Bertschinger and Natschläger, 2004; Shew et al., 2009) — and with the manuscript’s own §4.2 account of an inverted-U relationship between LC phasic/tonic-high activity and performance. EFT does not currently possess an analytic derivation of the γ–Q functional form from the master equation; the relationship between the latent gain parameter and the latent integrative state is an empirically motivated hypothesis external to the equation’s formal structure, and this limitation is now stated explicitly rather than resolved by assuming a single functional form.

A further clarification, sharpened in this revision, concerns exactly what empirical data can and cannot adjudicate. Because no measurement model presently links the observable pair (γ̂, PCI) to the latent pair (γ, Q) — see §5.1 and Table 1 — this manuscript does not claim that data can determine the functional form of the latent γ–Q relationship directly. What can be adjudicated empirically, and what Prediction P4 is accordingly designed to test, is the functional form of the observable γ̂–PCI relationship; a result at that level constrains, but does not by itself estimate, the corresponding latent γ–Q hypothesis. Earlier framings of P4 that referred to candidate models “relating γ̂ to Q” conflated these two levels; this revision corrects that conflation throughout.

Prediction P4 (§6.2) is accordingly reformulated as a model-selection problem among three explicit candidate functional forms relating the observable estimator γ̂ to the observable proxy PCI — not as a single presumed shape, and not as a direct test of the latent γ–Q relationship itself: M0 — the null model, no systematic relationship between γ̂ and PCI beyond noise; M1 — a monotonic, saturating (sigmoidal) relationship, in which PCI increases with γ̂ and plateaus at high values without decline; and M2 — a peaked (inverted-U) relationship, in which PCI rises with γ̂ toward a maximum near γc and then declines at supracritical γ̂ values, consistent with tonic-high LC-mediated degradation of signal-to-noise (Aston-Jones and Cohen, 2005; Sara and Bouret, 2012) and with peaked-computation accounts of criticality (Bertschinger and Natschläger, 2004; Shew et al., 2009). These three models are to be compared via BIC against empirical HRV–PCI data (§6.2, Prediction P4). Whichever model is favored constrains the corresponding hypothesis about the latent γ–Q relationship without establishing it directly, since that further inferential step still depends on a measurement model linking PCI to Q that does not yet exist. The Supplementary Figure S1 has been reconstructed accordingly to depict M0/M1/M2 as three explicit, visually distinguished candidate curves relating γ̂ to PCI — not to Q directly — with axes explicitly labeled to keep the latent construct Q, its proxy PCI, the latent construct γ, and its estimator γ̂ mutually distinct.

The candidate transition region γc is motivated by, but not established by, the neural-criticality literature: Beggs and Plenz (2003) demonstrated that cortical networks exhibit scale-free avalanche dynamics characteristic of systems near a phase transition; Shew and Plenz (2013) established that this critical regime is associated with maximal dynamic range and information transmission. Shine (2023) provided a candidate mechanistic account of how such a critical transition could be biologically implemented: diffuse neuromodulatory projections may function as intrinsic control parameters poising neurons near the critical point of a phase transition. None of these sources demonstrates that HRV-derived γ̂ is itself the control parameter governing cortical criticality; that specific claim is EFT’s own extension and is labeled Tier-2 throughout. The term ‘γc’ is retained for notational continuity with this literature, but the manuscript deliberately avoids asserting that γc is a true dynamical critical point until M1 versus M2 is adjudicated empirically (§6.2, Prediction P4b) — a distinction sharpened here in direct response to peer review, which correctly noted that ‘critical threshold’ presupposed more than the evidence currently supports.

A structural limitation of the master equation itself, identified through further review, is stated explicitly here rather than left implicit. Holding κ, αnk, g(ΔTnk), and β fixed, the first-order driving term κ·γ·Σαnk·g(ΔTnk) is monotonically increasing in γ, and the equation’s steady-state solution (Q = κγΣαnk·g(ΔTnk)/β) is accordingly monotonic in γ as well. The present equation, taken in this constant-parameter form, cannot by itself generate the descending limb of an inverted-U relationship (M2). Accordingly, if M2 were empirically favored under Prediction P4b — at the observable γ̂–PCI level — the present first-order phenomenological equation would require extension such that one or more effective parameters — most plausibly αnk, g(ΔTnk), or β — become state-dependent at supracritical γ (as indexed empirically by γ̂); the equation as currently specified does not generate this behavior on its own. This is stated as a consequence the model itself would face under one of its own candidate outcomes, not as a defect requiring immediate resolution: extending the equation before Prediction P4 has been tested empirically would be premature model elaboration.

Supporting the general plausibility that ongoing cortical dynamical regime is informative about PCI-indexed complexity — though not adjudicating between M1 and M2 — Maschke et al. (2024) demonstrated that EEG-derived criticality measures predict PCI values with a mean error below 7% across propofol, xenon, and ketamine anesthesia states. This finding is specific to the anesthetic-transition context studied; a bioRxiv preprint from the same group Newman et al. (2026) reports that in a heterogeneous cohort of patients with disorders of consciousness these criticality features did not generalize to predicting PCImax, and this boundary condition is noted here to avoid over-generalizing the finding beyond its tested domain. Whether autonomic coherence constitutes a modulatory input to cortical criticality at all remains to be tested directly within the EFT experimental program.

## 6 Empirical predictions and falsifiability

### 6.1 Falsifiability as a design criterion

The present section translates EFT’s temporal architecture, routing mechanisms, and master equation into five concrete, operationally defined empirical predictions (P1–P5, with P4 further divided into a minimal existence claim P4a and a conditional mechanistic-discrimination claim P4b) ordered by feasibility. Each prediction includes a specification of experimental design, primary operational variables, predicted direction and approximate effect size where estimable, and conditions under which the prediction would be considered falsified. All predictions are classified as Tier-2: derived from mechanistic reasoning grounded in established empirical literature, but their specific quantitative relationships have not been directly tested within EFT. These predictions concern probabilistic tendencies at the population level rather than deterministic effects within individuals. A single-subject (*N* = 1) pilot protocol referenced in earlier versions of this manuscript has been removed from this section entirely, in direct and more conservative response to Reviewer 1’s original request to remove or reframe it. On reflection, reframing it as a feasibility observation still risked implying methodological reliance on unreported data, which is inconsistent with the Data Availability Statement’s claim that no new empirical data are relied upon in this manuscript. No pilot data, outcomes, or design justification are referenced anywhere in this manuscript; feasibility of the study designs proposed below rests solely on the cited literature (e.g., Briand et al., 2020; Yap et al., 2020).

### 6.2 Empirical predictions

Convergent clinical evidence relevant to Prediction P1 is provided by Raimondo et al. (2017), who demonstrated in 127 patients with disorders of consciousness (vegetative state/unresponsive wakefulness syndrome, n = 70; minimally conscious state, n = 57) that cardiac autonomic markers — including stimulus-related cardiac-cycle modulations elicited during an auditory oddball paradigm — improved data-driven classification between the two diagnostic groups when combined with EEG complexity markers. This finding positions Prediction P1 as continuous with a demonstrated diagnostic phenomenon at the clinical extreme of the consciousness spectrum, and constitutes indirect empirical support for the general proposition that cardiac autonomic signals index functional differences in cortical integrative capacity. The clinical translation from group-level disorders-of-consciousness classification to healthy-population γ̂–PCI covariation under Prediction P1 requires direct experimental validation and is not assumed here; this is a Tier-2 anchor, not a demonstration of P1 itself.

### 6.3 Interpretive principles

Throughout Table 2, falsification criteria are stated, wherever feasible, using equivalence-testing logic (P1, P2, P3): a result counts as evidence against a prediction only when it demonstrates, with adequate statistical precision, that the true effect is smaller than a pre-registered minimal effect size of interest (SESOI). A non-significant or imprecise result that does not meet this standard is classified as inconclusive rather than as evidence against EFT. This distinction matters because a null *p*-value alone conflates two very different outcomes — a genuinely small or absent effect, and an underpowered or imprecise study — and only the former should count against a falsifiable claim. The same logic is extended to the model-comparison predictions (P4a, P4b) via the ΔBIC<4 inconclusive zone. This is a general principle of the EFT empirical program, not a case-by-case accommodation: absence of evidence is not treated as evidence of absence anywhere in Table 2.

**Table 2:** Summary of EFT empirical predictions.

Prediction	Design / Measures	Predicted effect	Falsification criterion
P1: HRV–PCI covariation	Within-subjects; 3 autonomic states; TMS-EEG; *n* ≥ 30	Positive γ̂–PCI association whose 95% CI excludes zero AND whose point estimate is ≥ the pre-registered minimal effect size (SESOI, *r* = 0.20); functional form adjudicated by P4a/P4b	Evidence against P1: a pre-registered equivalence test (TOST, region of equivalence to be specified in the registered protocol) supports the conclusion that the γ̂–PCI association is smaller than the SESOI (*r* = 0.20). A 95% CI that includes zero without also being precise enough to exclude the SESOI is classified as inconclusive (underpowered), not as evidence against P1
P2: tVNS → ΔTₚ markers	Double-blind sham crossover; ≥64ch EEG; ECG; *n* ≥ 40	↑P3b amplitude; ↑gamma synchrony; moderated by baseline γ̂; effect compatible with a pre-registered SESOI of *d* = 0.4	Evidence against P2: a pre-registered equivalence test (TOST, region of equivalence to be specified in the registered protocol) supports the conclusion that the true tVNS effect is smaller than *d* = 0.4, not merely a non-significant result. A result that is neither significant nor precise enough to exclude the SESOI is classified as inconclusive
P3: Stress → ↓ΔTᶠ	TSST vs. control; HRV and theta-gamma PAC at peak cortisol; cortisol, narrative coherence; *n* ≥ 35	↓PAC and ↓narrative coherence following acute stress, each of a magnitude meeting a pre-registered SESOI; mediated by γ̂	Evidence against P3: pre-registered equivalence tests (TOST, regions of equivalence to be specified in the registered protocol) support the conclusion that both the PAC and narrative-coherence effects are smaller than their respective SESOIs. A non-significant result on one or both measures without adequate precision to exclude the corresponding SESOI is classified as inconclusive, not as evidence against P3
P4a: Existence of a systematic γ̂–PCI relationship (minimal claim; constrains, but does not directly estimate, the latent γ–Q hypothesis)	Graded arousal states spanning subcritical to supracritical γ̂; BIC-based comparison of M1∪M2 (any systematic relationship) vs. M0 (null), all models defined over the observable pair (γ̂, PCI)	M1 or M2 outperforms M0 (ΔBIC≥4)	Three-way outcome, stated explicitly to avoid ambiguity: (i) systematic relationship — M1 or M2 outperforms M0 (ΔBIC≥4), supporting P4a; (ii) evidence against P4a — M0 is favored over both M1 and M2 by the same pre-specified threshold (ΔBIC≥4 in favor of M0), constituting positive evidence for the null rather than mere absence of a positive result; (iii) inconclusive — ΔBIC<4 in either direction, insufficient to favor any model. This is the minimal prediction on which EFT’s core claim depends
P4b: Mechanistic discrimination in the observable γ̂–PCI relationship (conditional on P4a)	Same dataset as P4a, conditional on P4a surviving; BIC-based comparison of M2 (peaked/inverted-U) vs. M1 (monotonic-saturating)	M2 outperforms M1 (ΔBIC≥4): supports the criticality/adaptive-gain interpretation of γc. M1 outperforms M2: EFT’s minimal claim (P4a) survives but the criticality-specific interpretation of γc does not.	Not independently falsifiable — this is a discrimination between two models that both already outperformed M0 under P4a; neither outcome speaks to the latent γ–Q relationship beyond what P4a already constrains
P5: SN disruption → selective ΔTᶠ impairment	cTBS AI/dACC; lesion studies; iEEG; *n* ≥ 25 per approach	↓PAC >> ↓P3b amplitude	No selective PAC reduction across ≥2 causal approaches

P1, P2, and P3 falsification criteria now uniformly distinguish evidence against a prediction (via pre-registered equivalence testing) from an inconclusive result, rather than treating any non-significant or imprecise finding as falsifying; P4 reformulated to test the observable γ̂–PCI relationship (constraining, but not directly estimating, the latent γ–Q relationship) and split into a minimal existence claim (P4a) and a conditional mechanistic-discrimination claim (P4b).

No single confirmatory result would establish EFT as a complete theory of consciousness; the cumulative pattern of evidence across predictions, evaluated against the master equation and temporal architecture, is the appropriate evidentiary standard. Individual variability in autonomic regulation, structural connectivity, and neuromodulatory baseline is expected and does not itself disconfirm EFT’s population-level predictions. Confirmatory evidence would strengthen EFT as a framework characterizing the physiological conditions associated with conscious access, but it would not resolve the hard problem of consciousness.

## 7 Limitations and boundary conditions

### 7.1 Scope and operationalisation

EFT is explicitly a Tier-2 theoretical framework: its mechanistic claims are grounded in and consistent with empirical literature, but the integrated routing pathway from autonomic coherence to cortical integration has not been tested as a unified circuit. No individual component of the master equation has been directly calibrated against empirical data; the specific values of κ, β, αnk, and the functional form of g(ΔTnk) remain undetermined. The normalized RMSSD-derived quantity γ̂ = RMSSD/RMSSD_basal is used as an operational estimator of γ but is a composite index influenced by respiratory rate, physical activity, pharmacological state, and cardiac pathology, and does not provide millisecond-resolved access to brainstem computational processes (Shaffer and Ginsberg, 2017; Task Force of the European Society of Cardiology and the North American Society of Pacing and Electrophysiology, 1996). PCI is used as an observational constraint on Q but is a perturbational measure that may not fully capture the moment-to-moment dynamics of spontaneous cortical integration. Theta-gamma PAC from scalp EEG provides only an indirect and spatially blurred index of hippocampal-cortical coupling.

The joint measurability of EFT’s three temporal regimes with a single method also remains an open methodological problem, noted here explicitly as a limitation rather than resolved by treating the regimes as empirically equivalent. EEG/MEG offers the temporal resolution required for ΔTᵢ and ΔTₚ but is largely restricted to cortical generators, without direct access to the proposed subcortical relay. fMRI can reach subcortical targets but its acquisition rate (1–2 s) and hemodynamic lag (4–6 s) are incompatible with the 50–500 ms timescales defined for ΔTᵢ/ΔTₚ, and would require high-field acquisition combined with demanding EEG–fMRI coupling protocols. HRV itself requires averaging windows of 30 s to 5 min — a discrepancy of at least three orders of magnitude with the 50–200 ms window defined for ΔTᵢ. Because of this, γ is explicitly defined as a tonic/baseline gain parameter that conditions the probability of transitions occurring on the much faster ΔTᵢ/ΔTₚ/ΔTᶠ timescales, rather than as an instantaneous multiplier operating within those windows — the two are different models with different measurable consequences, and EFT commits to the former (see also §5.1 on the quasi-static treatment of γ within each HRV estimation epoch). No claim is made that γ̂, the empirical estimator of γ, and the fast temporal regimes can currently be measured jointly with a single instrument; this is stated as a limitation of available methods, not resolved by the theory.

Additionally, translating tVNS protocols from controlled research environments to clinical settings presents specific challenges related to electrode placement standardization, individual variation in auricular anatomy, and the absence of validated biomarkers confirming target engagement at the NTS — issues comprehensively reviewed by Yap et al. (2020). As clarified in §4.1, this absence of a validated biomarker is particularly consequential for EFT because it also prevents dissociating NTS → PBN → LC engagement from NTS → VMb → insula engagement in current tVNS designs. These methodological constraints are pre-specified limitations that motivate the controlled multi-participant designs specified in §6.2 and Table 2.

### 7.2 Boundary conditions

EFT’s temporal architecture is developed primarily with reference to healthy adult human populations under standard laboratory conditions. The framework does not directly address without appropriate modification: infants and young children, whose neuromodulatory systems are undergoing rapid development; non-human animals, for whom the NTS-PBN-LC-SN routing pathway is conserved but phenomenological architecture cannot be assumed equivalent; clinical populations with disorders of autonomic regulation (vagal neuropathy, spinal cord injury) — for whom, as noted in Table 1, the mapping between raw RMSSD and the dimensionless γ̂ estimator may require population-specific recalibration rather than the healthy-adult baseline assumed elsewhere; individuals under pharmacological agents directly modulating the LC-NE system (atomoxetine, clonidine, propranolol); and states of anesthesia and disorders of consciousness, where temporal regimes may be disrupted at multiple points simultaneously.

### 7.3 The hard problem and EFT’s explanatory limits

EFT is a framework for the physiological conditions associated with conscious access. It does not claim to explain phenomenal consciousness—the subjective, qualitative character of experience—nor does it claim to resolve the hard problem. The three temporal regimes and the master equation characterize functional operations and their neurobiological correlates; they do not constitute an account of why these operations are accompanied by experience. This limitation is shared by all current neuroscientific frameworks for consciousness, including GNW, IIT, and PP/FEP.

## 8 Discussion and conclusion

The Emergent Flow Theory proposes that consciousness-supporting cortical integration is organized across three nested temporal regimes—ΔTᵢ (autonomic-interoceptive), ΔTₚ (conscious access), and ΔTᶠ (phenomenological-narrative)—and that the probability and quality of transitions between these regimes are modulated by a single integrative gain parameter: autonomic coherence (γ), a latent, dimensionless construct empirically estimated via a normalized HRV-derived estimator (γ̂). This claim is formalized in a dimensionally closed phenomenological dynamic equation for a latent integrated-complexity state (Q), explicitly distinguished from its observational proxy (PCI) in exact structural parallel to the γ/γ̂ distinction, and grounded in a multi-node routing pathway connecting ascending interoceptive afference — with the established NTS–VMb–insula relay and EFT’s own proposed NTS–PBN–LC axis now explicitly distinguished — through LC-NE gain modulation, Salience Network routing, CEN working-memory buffering, and DMN autobiographical integration, with a putative top-down feedback loop through the vmPFC now supported by direct human vmPFC–amygdala–HRV coactivation evidence (Thayer et al., 2012).

EFT makes four principal contributions to consciousness science. First, it identifies a specific, previously underspecified explanatory gap: the absence of a formal account of how autonomic and neuromodulatory state may dynamically modulate cortical integrative capacity across nested temporal windows. The claim that temporal architecture plays a constitutive—rather than merely correlative—role in conscious processing is shared with Northoff and Huang's (2017) temporo-spatial theory of consciousness (TTC), which places the brain’s intrinsic temporo-spatial organization at the center of its account of consciousness. EFT’s relationship to TTC is best specified along four points of difference rather than asserted as a single shared premise. (i) TTC asks how the brain’s own intrinsic temporo-spatial organization mediates dimensions of consciousness; EFT adopts the general premise that temporal structure is constitutive, but introduces an explicit physiological modulator external to that intrinsic cortical organization — autonomic coherence (γ) — as a further condition upon it. (ii) Where TTC characterizes temporo-spatial dynamics in general terms, EFT proposes a specific operational architecture of three nested processing windows (ΔTᵢ, ΔTₚ, ΔTᶠ) linked by an explicit gain equation. (iii) EFT ties this architecture to a specific gain parameter (γ) with a stated — if not yet derived — functional relationship to integrative capacity (§5.4), rather than leaving the temporo-spatial mediation unparameterised. (iv) EFT anchors its claims to manipulable, non-invasive observables (HRV, tVNS) and to falsifiable predictions (P1–P5) with explicit null-result criteria, providing an empirical route that is not the primary focus of TTC’s more general theoretical account. EFT should therefore be understood as orthogonal and complementary to TTC rather than as a replacement or a special case of it: TTC specifies the intrinsic temporo-spatial organization proposed to support consciousness, whereas EFT asks whether the accessibility and stability of that organization are conditioned by autonomic gain. Second, it proposes a tractable, dimensionally closed dynamic model that organizes this account in falsifiable form, identifying specific parameters—γc, β, αnk, and g—that future experimental work should constrain. Third, it generates five concrete, operationally defined empirical predictions (P1–P5), each with explicit adjudication criteria — P4 comprising a falsifiable existence test (P4a) followed by conditional model discrimination (P4b) — including a model-selection prediction that adjudicates among competing functional forms of the observable γ̂–PCI relationship — constraining, though not directly estimating, the latent γ–Q relationship — rather than assuming a single functional form in advance. Notably, the master equation in its present first-order form generates only the monotonic M1 profile; should P4b instead favor M2, the equation itself — not merely its interpretation — would require extension (e.g., state-dependence of αnk, g(ΔTnk), or β at supracritical γ). Fourth, it provides a conceptually integrative account complementary to GNW, IIT, and PP/FEP, situating autonomic coherence as a proposed physiological precondition within which the integrative capacities described by these frameworks may be realized.

EFT is offered as a Tier-2 theoretical framework: grounded in a substantial body of empirical literature, mechanistically coherent, and formally specified, but requiring direct experimental validation of its integrated claims before its status can be elevated. The empirical program it generates—combining psychophysiology, TMS-EEG, high-density EEG, non-invasive neuromodulation, and causal disruption paradigms—is achievable with current methodological capabilities. If the predicted relationships between autonomic coherence, neuromodulatory gain, and the temporal architecture of cortical integration are confirmed, EFT will have provided substantive empirical support for a functional bridge between the body’s regulatory physiology and the temporal dynamics of conscious access—a bridge whose elaboration, we argue, is necessary for any complete account of the conditions under which conscious experience arises.

Convergent evidence at multiple levels of analysis supports the theoretical architecture advanced here: ascending neuromodulatory gain modulation drives whole-brain information-processing transitions at intermediate gain values, providing a systems-level precedent for the candidate transition region EFT denotes γc (Li et al., 2019; Shine, 2023); phasic neuromodulatory bursts mediate abrupt network reconfigurations coinciding with perceptual state transitions (Wainstein et al., 2025); and cardiac autonomic markers improve consciousness classification in disordered-consciousness populations beyond cortical complexity metrics alone (Raimondo et al., 2017) — providing mechanistic, behavioral, and clinical anchors for the autonomic–cortical integration hypothesis EFT formalizes. None of this convergent evidence directly tests EFT’s specific claims; each anchor is offered at the Tier-2 level of plausibility and motivation, not confirmation.

## Data Availability

The original contributions presented in the study are included in the article/Supplementary material, further inquiries can be directed to the corresponding author.
