# Supplementary material for: Emergent flow theory: autonomic coherence as a gain parameter for the temporal architecture of conscious integration
Source: Front Integr Neurosci. 2026 Sep 18;20:1841714. doi: 10.3389/fnint.2026.1841714 (PMC13631055; doi:10.3389/fnint.2026.1841714)
Supplement: Supplementary file 1 [file Data_Sheet_1.PDF]

SUPPLEMENTARY MATERIAL

PREFATORY DISCLAIMER: EPISTEMIC STATUS OF THIS SUPPLEMENTARY MATERIAL

The sections that follow constitute strictly delimited Tier-3 theoretical extensions of EFT. They are exploratory in character and are provided as a disciplined research horizon only.

Four epistemic clarifications — read before proceeding

These sections are exploratory theoretical extensions only. They do not form part of the empirically testable core of EFT.

They are not required for the falsifiability or internal coherence of the main EFT model. The master equation $dQ/dt = \kappa \cdot \gamma \cdot \Sigma(n,k)[\alpha nk \cdot g(\Delta Tnk)] - \beta Q + \xi(t)$ stands fully independently of any content presented here. γ denotes the dimensionless, latent effective autonomic-coherence parameter formalised in Table 1 of the main manuscript, exactly as Q is distinguished from its observational proxy PCI; $\hat{\gamma}$ = RMSSD/RMSSD_basal is its candidate operational estimator — one normalisation currently in use, not frozen as the unique or final operationalisation of γ — used to calibrate and test the equation's predictions but not written into the equation itself.

They do not alter the empirical core built around ΔT_i, ΔT_p, and ΔT^f, nor do they modify the probabilistic routing architecture described in the main manuscript or its associated operational markers.

They are offered only as a future research programme, subject to empirical adjudication by methods not yet available or sufficiently developed.

For the avoidance of doubt: the Tier-3 extensions presented in this Supplementary Material do not modify the master equation, are not required for the internal coherence or falsifiability of EFT's core empirical predictions (P1–P5), and are provided solely as a disciplined research horizon subject to future empirical adjudication. Reviewers and readers are advised to evaluate the main manuscript entirely independently of this material.

SUPPLEMENTARY SECTION S1 — SCOPE AND EPISTEMIC STATUS OF TIER-3 EXTENSIONS

The empirical and theoretical core of EFT is grounded in well-characterised, experimentally accessible phenomena: the relationship between cardiac autonomic regulation as indexed by HRV, locus coeruleus–norepinephrine (LC-NE) gain modulation, Salience Network routing, Central Executive Network working-memory buffering, and Default Mode Network autobiographical integration. The operational markers associated with this core—including RMSSD and HF-HRV, the P3b event-related potential component, broadband gamma-band synchrony, the Perturbational Complexity Index (PCI), and theta–gamma phase-amplitude coupling (PAC)—are measurable with techniques available to contemporary systems neuroscience laboratories.

The present Supplementary Material ventures beyond this empirically grounded core to consider whether EFT's temporal architecture might, in principle, articulate with phenomena operating at spatial and temporal scales below those captured by the main text. The following sections address four candidate

domains organised as a speculative descending hierarchy: (i) the microphysical and geometric boundary conditions that may constitute the deepest physical context for biological information integration (§S2); (ii) fast bioelectrical and intracellular coordination mechanisms operating below the timescale of the ΔT_i regime (§S3); (iii) slower biochemical and epigenetic processes that may constrain the long-term baseline on which the ΔT_i–ΔT_p–ΔT^f architecture operates (§S4); and (iv) the explicit relationship between these Tier-3 extensions and EFT's master equation parameters (§S5).

Three organising epistemic principles govern the presentation throughout. First, a strict terminological discipline is observed: claims are explicitly labelled as speculative, plausible, hypothetical, or established, and these designations are not interchangeable. Second, no inference is drawn from exploratory proposals to modifications of the master equation or to the empirical predictions of the main text. Third, the material presented here is not offered as a foundation for any form of quantum consciousness or mystical interpretation of EFT; §S6 provides explicit demarcation.

S1.1 EFT in the Landscape of Contemporary Theories of Consciousness

EFT's account of conscious processing as the product of hierarchically nested temporal integration modulated by autonomic coherence (γ) operates in dialogue with several influential frameworks in contemporary consciousness science. This section briefly situates EFT relative to these frameworks to clarify points of convergence and demarcation, without subsuming EFT within any of them.

Damasio's organism-centred framework (1999, 2010) identifies a hierarchy of self-processes—proto-self, core self, and autobiographical self—anchored in continuous interoceptive signalling from the body to the brainstem and cortex. EFT's autonomic coherence parameter γ, for which HRV-derived $\hat{\gamma}$ provides a candidate operational estimator, is functionally compatible with the proto-self level of this hierarchy: it represents the ongoing visceral reference that the organism maintains about its internal state, which in EFT is proposed to modulate the conditions for hierarchical cortical integration. This alignment is treated as a functional convergence, not an ontological identification.

Northoff's temporo-spatial theory of consciousness (TTC; Northoff & Huang, 2017) proposes that the brain's intrinsic temporal and spatial organisation constitutes a necessary scaffold for conscious experience. The convergence with EFT's $\Delta T_i/\Delta T_p/\Delta T^f$ temporal hierarchy is substantive: both frameworks assign constitutive, not merely correlative, status to the temporal structure of neural activity. The distinction is that EFT introduces an explicit autonomic regulator (γ) operating on this temporal hierarchy, a parameter not formalised in Northoff's framework. The two theories are best understood as independently convergent rather than competing.

Within the Free Energy Principle (FEP; Friston, 2010) and its extensions in active inference (Friston et al., 2017), neuromodulatory systems regulate the precision—or inverse variance—assigned to ascending prediction errors within hierarchical generative models. Barrett and Simmons (2015) extended this within a predictive coding framework for interoceptive signals. EFT's autonomic coherence parameter γ can be interpreted within this framework as a physiological contributor to precision regulation at the level of ascending interoceptive inference—a reading that is consistent with Tier-2 EFT and requires no Tier-3 extensions (Feldman & Friston, 2010; Yu & Dayan, 2005).

S1.2 Visual Phototransduction as a Comparative Temporal Analogy (Tier-2)

Visual phototransduction provides a well-characterized biological cascade in which an ultrafast molecular event is converted into progressively amplified neural signals across multiple temporal scales. The following account describes three phases of this cascade and their comparative temporal correspondence to EFT's conceptual architecture. All cross-regime alignments are Tier-2 (comparative temporal

correspondence only; not mechanistic equivalence). This analogy is referenced from the main manuscript (§3.1) and is retained here as its sole location, following the removal of a redundant shorter version from the main text's Limitations section during a previous revision for parsimony.

Phase 1 — Ultrafast Molecular Transduction (~200 fs)

[Tier-1] Photon absorption by rhodopsin induces isomerization of the 11-cis retinal chromophore into its all-trans configuration within approximately ~200 femtoseconds (Schoenlein et al., 1991). This process involves ultrafast excited-state and vibrational dynamics directly resolved by femtosecond spectroscopy.

[Tier-2] EFT alignment: This event is temporally analogous to the conceptual ΔTq regime — a speculative ultrafast threshold discussed in §S2.1. This correspondence is strictly temporal and does not imply mechanistic equivalence or validation of any subcellular EFT mechanism, nor does it provide support for Orch-OR or quantum coherence models in neural tissue.

Phase 2 — Biochemical Amplification (~1–10 ms)

[Tier-1] The initial isomerization triggers a G-protein-mediated amplification cascade: activated rhodopsin couples to transducin, activating phosphodiesterase and leading to cGMP hydrolysis and channel closure. A single photoisomerization event can suppress current through hundreds of channels, demonstrating biological amplification of localized molecular events (Baylor et al., 1979).

[Tier-2] EFT alignment: This amplification phase is temporally analogous to the conceptual ΔTbio-fast regime. The correspondence is organizational and temporal only; it does not imply mechanistic identity between phototransduction biochemistry and any proposed intracellular mechanism within EFT.

Phase 3 — Early Subcortical Transmission (early tens of ms)

[Tier-1] Retinal ganglion cells project to the lateral geniculate nucleus (LGN) via the primary image-forming visual pathway, and to the superior colliculus via a parallel pathway subserving visuomotor functions. Retinal output reaches subcortical targets on early tens-of-milliseconds timescales depending on pathway characteristics and stimulus conditions (Sherman & Guillery, 2002).

[Tier-2] EFT alignment: This early subcortical transmission phase is temporally compatible with the transition toward ΔT_i (~50–200 ms), without implying shared anatomy or mechanism.

> *DEMARCATION: The visual pathway (retina → LGN → cortex) and the autonomic-interoceptive pathway (vagus → NTS → PBN → LC → thalamus → insula) are anatomically distinct and functionally parallel systems. Any convergence occurs at higher-order integrative networks — including insular cortex — rather than through a single continuous anatomical chain. This comparative analogy does not assert anatomical continuity between these pathways.*

Table S1. Phototransduction Phases — EFT Temporal Alignment

Phase	Timescale	Biological Event	EFT Alignment (Tier-2)
1 — Ultrafast	200 fs	Retinal isomerization (rhodopsin)	ΔTq — temporal analogy only; not mechanistic validation
2 — Amplification	1–10 ms	G-protein cascade; cGMP hydrolysis; channel closure	ΔTbio-fast — organizational analogy only

Phase	Timescale	Biological Event	EFT Alignment (Tier-2)
3 — Subcortical	Early tens of ms	RGC output to LGN / superior colliculus	Transition toward ΔT_i — parallel pathway; distinct anatomy

S1.3 Candidate Reticulo-Thalamic Transitional Sub-window (ΔTs, Tier-2)

[Tier-2] DEFINITION AND EPISTEMIC STATUS: In the current version of EFT, the transition from early subcortical integration (ΔT_i) to conscious access (ΔT_p) may involve a candidate reticulo-thalamic modulatory sub-window, here provisionally designated ΔTs (~160–200 ms). This interval is introduced not as a formally established regime of the model, but as a Tier-2 exploratory refinement. It designates a hypothetical late transitional segment within or at the upper boundary of ΔT_i, in which arousal-related gain control, thalamocortical coordination, and late preconscious integration may contribute to signal stabilization before conscious access. ΔTs does not replace ΔT_i and does not modify EFT's master equation in any form.

[Tier-2] MECHANISTIC RATIONALE: This proposal is motivated by convergent evidence indicating that the locus coeruleus–norepinephrine system modulates adaptive gain and signal-to-noise ratio across thalamocortical circuits (Aston-Jones & Cohen, 2005), and that thalamocortical dynamics regulate information flow under changing arousal states (Sherman & Guillery, 2002). The ascending reticular activating system (ARAS) and its thalamic relay nodes may constitute a neuroanatomical substrate through which autonomic coherence (γ) influences accessibility of the ΔT_p ignition threshold. As clarified in the main manuscript (§4.1), the anatomical evidence for ascending LC-directed gain-setting pathways is rodent tract-tracing evidence (Kawai, 2018), not primate or human evidence, and the ΔTs proposal inherits the same evidentiary caution.

[Tier-2] Consistent with Buzsáki's (2006) oscillatory syntax framework, the critical coincidence window proposed for ΔTs aligns with the temporal scale at which fast-spiking parvalbumin-positive interneurons generate local gamma-band synchrony and phase-locked neural assemblies. Within this hypothesis, PAC-mediated temporal coordination is considered a candidate mechanism by which temporally dispersed signals could achieve sufficient coincidence for subsequent access to ΔT_p. The N200 is proposed as a candidate electrophysiological marker of this transitional interval, rather than as a unique signature of the underlying mechanism. This mechanistic elaboration is Tier-2: the specific chain from autonomic coherence (γ) through LC–NE modulation to PV interneuron gamma synchrony at ΔTs has not been validated as a unified circuit in conscious humans and requires direct empirical investigation (Buzsáki & Draguhn, 2004; Buzsáki, 2006).

> *The full mechanistic, empirical, and theoretical programme for ΔTs — including its relationship to the N200 electrophysiological signature, the Striatal Beat Frequency mechanism, and the Buzsáki oscillatory syntax framework — remains an open direction for future development and is not elaborated further here.*
>
> *DEMARCATION: EFT does not currently claim that gamma synchronization, locus coeruleus discharge, or thalamic resonance alone explain the binding problem. ΔTs does not claim to resolve the longstanding debate over the role of gamma oscillations in conscious integration (Crick & Koch, 1990; Singer & Gray, 1995), which remains empirically and theoretically unresolved. ΔTs is offered as an exploratory organizational construct to guide future empirical work, not as a validated component of EFT's empirical programme.*

SUPPLEMENTARY SECTION S2 — SPECULATIVE MICROPHYSICAL BOUNDARY CONDITIONS

S2.1 Physical Constraints on Biological Information Integration and the ΔTq Boundary

All biological information processing is ultimately constrained by physical principles governing energy transfer, molecular dynamics, and thermodynamic limits. In neural systems, these constraints are manifested through membrane potentials, ion channel kinetics, and synaptic transmission, which collectively determine the timescales and energetic costs of information integration. Attwell and Laughlin (2001) demonstrated that neural signalling is metabolically expensive, with a substantial fraction of the brain's energy budget devoted to maintaining ion gradients and synaptic activity. This energetic constraint provides a physiological context for EFT's phenomenological dissipation term β in the master equation, without identifying β with a specific metabolic process: integrated cortical states are thermodynamically costly and decay toward baseline in the absence of sustained driving input.

At the molecular level, energy transport within proteins and cytoskeletal structures involves vibrational energy transfer mechanisms propagating through covalent bonding networks. Leitner (2008) and Leitner et al. (2019) characterised how vibrational excitations—describable as phonon-like modes in protein scaffolds—propagate via anharmonic coupling in patterns reflecting the protein's topology and dynamical architecture. These dynamics operate on sub-picosecond to nanosecond timescales and are, strictly speaking, classical thermal phenomena amenable to quantum statistical mechanical description, without invoking quantum coherence in any computationally relevant sense.

[Tier-3] Within a strictly speculative Tier-3 frame, one might ask whether the ensemble properties of such vibrational transport—averaged across the thousands of protein molecules constituting a single synapse—could conceivably contribute to the stochastic background dynamics represented by ξ(t) in EFT's master equation. This is a hypothetical possibility, not a demonstrated relationship, and it does not modify the equation's current formulation. The concept of ΔTq is introduced here as a speculative microphysical boundary condition: a conceptual placeholder for microphysical timescales below those represented in EFT's current biological architecture. This proposal is not required for EFT's falsifiability and should be understood strictly as a research horizon marker.

S2.2 A Geometric Heuristic: Twistor Theory as a Non-Mechanistic Analogy

[Tier-3] Twistor theory (Penrose, 1967) is retained here solely as a geometric heuristic for future formal work on constrained state accessibility, not as a physical or mechanistic claim about neural tissue. In relativistic geometry, causal structure distinguishes events that are reachable under physical propagation constraints from those that are not. EFT does not equate this causal structure with neural dynamics, nor does it claim that biological systems implement twistor geometry. The analogy is narrower: future formulations of integrative accessibility may require a geometry that distinguishes dynamically reachable from unreachable regions of state space under physiological and stochastic constraints. Whether twistor-inspired mathematics offers any useful formal vocabulary for such a problem is entirely open, and no element of the present EFT model depends on this possibility. This geometric analogy is retained as a conceptual placeholder for future work on constrained state-space accessibility and stochastic reachability, neither of which is formalised in the present manuscript.

S2.3 Autonomic Coherence as Precision Weighting: Integration with the Free Energy Principle

Within the Free Energy Principle (FEP; Friston, 2010) and its extensions in active inference (Friston et al., 2017), neuromodulatory systems regulate the precision—or inverse variance—assigned to ascending prediction errors within hierarchical generative models. The LC-NE system has specifically been linked to the global gain of ascending prediction-error signals (Feldman & Friston, 2010; Yu & Dayan, 2005).

[Tier-2] EFT's autonomic coherence parameter γ, estimated operationally through the candidate HRV-derived estimator $\hat{\gamma}$ (formally defined in Table 1 of the main manuscript), can be interpreted within this framework as a physiological contributor to precision regulation at the level of ascending interoceptive inference. Through the ascending interoceptive pathways discussed in the main manuscript (§4.1) — including EFT's proposed NTS–PBN–LC axis, classified there as an organisational hypothesis grounded in rodent tract-tracing evidence (Kawai, 2018) rather than a directly demonstrated human circuit — ascending visceral signals are proposed to influence noradrenergic tone, which in turn modulates cortical signal-to-noise ratios and the probability of global ignition events consistent with the ΔT_p regime. This reading of γ as a physiological precision-weighting parameter is consistent with Tier-2 EFT and requires no quantum or subcellular hypotheses.

At the formal level, the concept of the Markov blanket (Friston et al., 2020) offers a useful analogy for describing how an organism maintains a coherent boundary between its internal states and external perturbations. In EFT, the Markov blanket is used only as a formal analogy for organismic boundary maintenance; the nested windows $\Delta T_i/\Delta T_p/\Delta T^f$ specify the temporal structure within which such boundary-sensitive integration unfolds, with γ functioning as the autonomic regulator that modulates this boundary's integrative capacity. This reading does not redefine EFT in terms of the FEP, nor does it introduce free energy minimisation as a mechanistic component of the model.

[Tier-3] At the Tier-3 speculative level only, one might ask whether the reliability of ascending interoceptive signals has substrate determinants extending to the molecular dynamics characterised in §S2.1. This proposal is entirely hypothetical and is mentioned solely as a candidate direction for future theoretical integration.

S2.4 The Poised Realm, Neural Criticality, and Epistemic Demarcation

[Tier-3] Kauffman (2016, 2019) proposed the concept of the Poised Realm to describe systems operating near the classical–quantum boundary. This concept is offered here strictly as conceptual vocabulary for characterising a class of dynamical regime—systems near phase transitions—and not as a claim about quantum coherence in neural tissue. The relevance to EFT is indirect and limited to the following observation: the main manuscript (§5.4, §6.2) now formulates the observable $\hat{\gamma}$–PCI relationship — which constrains, but does not directly estimate, the latent γ–Q relationship — as an open empirical question adjudicated among three competing models (M0: no relationship; M1: monotonic-saturating; M2: peaked/inverted-U), rather than asserting a single sigmoidal form centred on a confirmed critical threshold. The Poised Realm concept, stripped of any quantum-computational implication, provides a loose conceptual analogy for describing systems that amplify microscopic fluctuations into macroscopic state transitions through nonlinear dynamics near phase boundaries — a class of dynamics broadly consistent with, but not confirmatory of, EFT's candidate transition region γc, whichever of M1 or M2 the data eventually favour.

> *DEMARCATION: EFT does not endorse the Orchestrated Objective Reduction (Orch-OR) hypothesis (Hameroff & Penrose, 1998). EFT does not endorse any form of quantum consciousness claim. No mystical, vitalistic, or non-*

falsifiable interpretation of the proposals in this section is warranted or intended. None of the mechanisms discussed in this section modify EFT's master equation.

SUPPLEMENTARY SECTION S3 — ΔT_BIO-FAST: BIOELECTRICAL AND SUBCELLULAR COORDINATION

S3.1 Defining the Speculative Temporal Regime

EFT's smallest operationally defined temporal regime, ΔT_i, operates at approximately 50–200 ms. Below this window lies a range of biological coordination phenomena operating at timescales from microseconds to tens of milliseconds: action potential propagation, dendritic integration of excitatory and inhibitory postsynaptic currents, gap-junction-mediated electrical coupling, and potential non-synaptic signalling mechanisms. This section considers whether these phenomena might constitute a 'ΔT_bio-fast' domain providing a substrate for fast biological coordination below the temporal resolution of EFT's current architecture.

This section is fully speculative and presented as an exploratory proposal only. No component of EFT's master equation is modified, and no empirical prediction of the main text is contingent on the mechanisms discussed here.

S3.2 Bioelectric Signalling as Distributed Cellular Coordination

Michael Levin and colleagues have developed an extensive research programme characterising the role of bioelectric signals—membrane voltage gradients, gap-junction-mediated ionic communication, and endogenous electric fields—in the coordination of cellular behaviour during development, regeneration, and pattern formation (Levin, 2012, 2019; Levin & Martyniuk, 2018). This work constitutes a legitimate and empirically grounded framework for understanding distributed cellular coordination that does not depend on synaptic transmission and that operates across timescales from seconds to developmental epochs.

[Tier-3] Relative to EFT's ΔT_i regime (milliseconds to seconds), Levin's framework is relevant as a speculative conceptual scaffold for the following reason: if bioelectric coordination mechanisms operating across populations of neurons and glial cells were shown to influence neural dynamics at timescales relevant to or preceding ΔT_i, they could conceivably contribute to the establishment of rapid field effects that influence the initial gain conditions of the ΔT_i regime. This remains entirely hypothetical. Critically, Levin's programme has been developed primarily in non-neural tissue and developmental biology; its extension to conscious integration in the adult human brain requires empirical work that has not yet been conducted.

S3.3 Solitons, Phonons, and Intracellular Oscillatory Dynamics

[Tier-3] Theoretical proposals for solitonic and phononic signalling in biological membranes and cytoskeletal structures (Heimburg & Jackson, 2005; Tuszynski et al., 1995; Craddock et al., 2014) remain empirically contested; direct evidence for their functional role in neural computation is limited, and no parameterisation within EFT's current framework is possible. They are noted here only for completeness.

A more empirically established mechanism for fast subcellular coordination in neural tissue is electrical coupling via gap junctions, which enables direct ionic communication between neurons on millisecond timescales without chemical synaptic transmission (Connors & Long, 2004). This mechanism, unlike solitonic proposals, has well-documented functional roles in cortical interneuron synchronisation and may constitute a more parsimonious substrate for the fast coordination dynamics hypothesised in this section.

S3.4 Biophotons as a Hypothetical Auxiliary Phenomenon

[Tier-3] Ultra-weak photon emission has been documented in biological and neural tissue, and speculative proposals have considered possible roles in neural signalling (Tang & Dai, 2014). No evidence presently establishes a necessary or sufficient role in conscious integration. EFT therefore does not incorporate biophotons mechanistically; they are noted solely as a Tier-3 auxiliary possibility.

S3.5 Mesoscopic Vibrational Energy Transport as a Candidate Contributor to ξ(t)

At the mesoscopic level, protein complexes and macromolecular networks within neural tissue exhibit collective vibrational modes that can be formally described as phonon-like excitations within highly coupled biomolecular systems (Leitner, 2008; Leitner et al., 2019). These dynamics reflect anisotropic, topology-constrained energy transport governed by nonlinear coupling interactions—phenomena fully accountable within classical statistical mechanics and nonequilibrium thermodynamics, without invoking macroscopic quantum coherence or computationally relevant quantum effects at timescales incompatible with thermal decoherence in physiological tissue.

[Tier-3] Within EFT's formal architecture, such mesoscopic vibrational dynamics could, in principle, contribute to sources of intrinsic variability that are coarse-grained phenomenologically into ξ(t) in the master equation. This interpretation does not introduce a new level of computation into EFT's architecture, nor does it alter the master equation in its current form. All claims in this section are Tier-3 and strictly delimited to the domain of classical biophysics.

SUPPLEMENTARY SECTION S4 — ΔT_BIO-SLOW: CELLULAR MEMORY AND BIOLOGICAL INERTIA

S4.1 The Concept of Biological Inertia in EFT

EFT's master equation characterises the moment-to-moment dynamics of Q as a function of γ, αnk, g(ΔTnk), β, and ξ(t). What the main text does not address is what determines the long-run baseline around which these parameters fluctuate—a question implicating processes operating at timescales from hours to years. This section introduces the speculative concept of 'ΔT_bio-slow' to denote the class of biological processes that may constrain the effective baseline of EFT's parameters. The candidate mechanisms considered here—biochemical consolidation, RNA-mediated synaptic persistence, and epigenetic modification—are presented as a plausible theoretical extension, not as established components of EFT's framework.

S4.2 Cellular Memory and Biochemical Consolidation

Kukushkin and Carew (2017) developed a framework for cellular memory addressing how individual neurons process temporal patterns of stimulation and generate persistent changes in their responsiveness through a hierarchical system of molecular and cellular time windows. Their work demonstrates that the temporal structure of neural activation—not merely its magnitude—determines the nature and persistence of synaptic modification, a finding directly relevant to EFT's claim that temporal integration windows are constitutive of conscious processing.

[Tier-3] From EFT's perspective, a Tier-3 extension might propose that chronic patterns of autonomic regulation—represented in the long-run distribution of γ values over weeks and months—could influence the molecular state of neurons in the NTS-PBN-LC axis, ultimately contributing to slow changes in effective

coupling parameters represented phenomenologically by αnk for specific node–regime pairs. This is a speculative mechanistic proposal that has not been tested within EFT's framework.

S4.3 RNA-Mediated Synaptic Persistence

A growing literature documents the role of RNA-mediated mechanisms in the persistence of synaptic changes. Work from multiple groups has characterised how local translation of synaptically localised messenger RNA contributes to the maintenance of long-term potentiation and synaptic tagging (Martin & Kosik, 2002). Investigations of prion-like RNA-binding proteins—including CPEB and its homologues—have demonstrated that self-perpetuating molecular states may contribute to the very long-term persistence of synaptic modifications (Si, Lindquist, & Kandel, 2003).

[Tier-3] In a strictly speculative register, self-perpetuating molecular states in the synaptic proteome may constitute a molecular substrate for the relative stability of αnk values posited in the main text—a history-encoding property that could, in principle, be shaped by sustained changes in autonomic state through the ΔT_bio-slow pathway. This connection is currently unparameterised.

S4.4 Epigenetic Modification, Allostasis, and Long-Run Baseline Constraints

Epigenetic mechanisms—including DNA methylation, histone modification, and chromatin remodelling—regulate gene expression in neural circuits over timescales from hours to the lifetime of the organism (Nestler, 2014). Nestler's work on epigenetic stabilisation of neural states demonstrates that chronic patterns of neural activity leave durable molecular traces that constrain future responsiveness of the same circuits.

[Tier-3] Within the Tier-3 framework, the long-run distribution of γ values could speculatively drive epigenetically mediated changes in the molecular architecture of EFT's routing nodes, establishing a biological inertia in the direction of either facilitated or constrained cortical integration.

McEwen's work on allostasis and biological inertia provides the physiological grounding for this proposal. The HPA axis's glucocorticoid response, through its effects on hippocampal neurogenesis and dendritic morphology (McEwen, 2007), constitutes an empirically characterised pathway through which chronic physiological states alter the structural substrate of cognitive function. The concept of allostatic load (McEwen & Wingfield, 2003) describes the cumulative biological cost of sustained deviation from optimal autonomic regulation—analogous, within EFT's speculative framework, to sustained shifts in the long-run distribution of γ toward ranges associated with reduced integrative capacity. Arnsten's documentation of stress-induced weakening of prefrontal networks (Arnsten, 2015) provides a further empirical anchor: the same chronic glucocorticoid exposure that McEwen characterises at the cellular level produces the measurable prefrontal connectivity deficits that Arnsten characterises at the circuit level, both conceptually consistent with conditions that could reduce the capacity of executive networks to sustain integration relevant to ΔT_p.

> *Important clarification: This framework does not imply that trauma, clinical depression, or psychotherapeutic outcomes are fully explained by these mechanisms alone, or that they reduce to autonomous ΔT_bio-slow dynamics without the involvement of complex psychosocial and developmental processes.*
>
> *SCOPE: an earlier version of this note referenced a formal relationship to 'ΔTv' and pointed to a dedicated manuscript in preparation. That forward reference, and any formalisation of stochastic-reachability or accessibility-cone constructs building on ΔT_bio-slow, remains outside the scope of both the main manuscript and this Supplementary Material. Consistent with the main programme's decision to keep speculative mathematical extensions out of material currently under peer review, no such formalism is introduced here or elsewhere in this document.*

SUPPLEMENTARY SECTION S5 — RELATION TO EFT VARIABLES WITHOUT MODIFYING THE MASTER EQUATION

S5.1 The Inviolable Boundary of the Master Equation

The exploratory extensions in §S2–S4 do not modify, extend, or reinterpret the master equation of EFT as presented in the main text:

$$dQ/dt = \kappa \cdot \gamma \cdot \Sigma(n,k)\ [\alpha nk \cdot g(\Delta Tnk)] - \beta Q + \xi(t)$$

This equation stands as a complete phenomenological model in its Tier-2 form, with γ denoting the dimensionless, latent effective autonomic-coherence parameter and Q the latent integrated-complexity state, both as formally defined in Table 1 of the main manuscript; $\hat{\gamma}$ is the empirical estimator of γ used for calibration and testing (§6 of the main manuscript) but does not appear as a literal term in the equation itself, exactly as PCI does not appear as a literal term despite operationalising Q. No parameter is added, removed, or reinterpreted on the basis of Tier-3 material. This boundary is not arbitrary—the master equation is the formal core of EFT's scientific programme, and its integrity must not be compromised by incorporating speculative extensions before those extensions have received independent empirical support.

S5.2 Possible Directions for Future Parameter Elaboration

Without modifying the current formulation, two directions remain genuinely open for future theoretical work; a third, noted in earlier versions of this Supplementary Material, has since been partially addressed in the main manuscript and is updated accordingly below.

First, a future extension might model αnk as a slowly evolving variable shaped by long-run cellular memory and epigenetic modification described in §S4—requiring empirical demonstration that the relevant molecular mechanisms contribute to connectivity variation at macroscopic scales relevant to EFT.

Second, this Supplementary Material previously proposed, as a future direction, decomposing γ into a dynamic component and a trait-level baseline component indexed by long-run RMSSD metrics. This distinction is now represented at the measurement level in the main manuscript (§5.1, Table 1): γ is retained as the latent theoretical gain parameter entering the master equation, while $\hat{\gamma}$ = RMSSD/RMSSD_basal provides its candidate normalised operational estimator, calibrated against an individual's own resting baseline. This does not constitute a decomposition of γ within the master equation itself — $\hat{\gamma}$ remains outside the equation, exactly as PCI does relative to Q. What remains open, and is not yet addressed, is whether the baseline term itself (RMSSD_basal) should be modelled as a slowly drifting variable shaped by the ΔT_bio-slow mechanisms discussed in §S4, rather than treated as a fixed per-individual constant. This narrower question is offered as the updated future direction.

Third, for current operational modelling, ξ(t) may be represented as additive Gaussian noise; EFT does not thereby claim that the underlying biological fluctuations are themselves Gaussian or generated by a single mechanism. Any modification of its mathematical form on the basis of subcellular mechanisms discussed in §S2–S3 would be entirely hypothetical and currently unparameterised. These potential directions are offered as a research horizon only and do not constitute predictions of EFT in its current form.

S5.3 The Self in EFT: A Temporally Grounded, Non-Ontological Formulation

The Tier-3 extensions reviewed above, taken together with the Tier-2 framework of the main text, permit a coherent formulation of selfhood that is consistent with contemporary neuroscience and avoids

ontological overcommitment. In EFT, the functional self can be described as a dynamic organism-level inference the organism maintains about the causal boundaries between its internal states and the external world. This inference is sustained by autonomic coherence (γ), structured temporally by the nested integration windows ($\Delta T_i/\Delta T_p/\Delta T^f$), and delimited informationally by a boundary analogous to the Markov blanket. This formulation converges with Damasio's proto-self homeostatic process (1999), the intrinsic temporal structure of neural activity identified by Northoff and Huang (2017), and the biological inertia of the autonomic system characterised by McEwen (2007)—without committing to strong ontological claims about the nature of subjective experience.

This formulation is intentionally restrained. It does not resolve the hard problem of consciousness, which EFT explicitly acknowledges as an open philosophical question beyond its empirical scope. It does not identify the self with any single neural structure or neuromodulatory parameter. It claims only that the organism's capacity to maintain a coherent, temporally integrated representation of the boundary between self and world is a candidate functional condition for the forms of conscious access that EFT's main framework characterises.

SUPPLEMENTARY SECTION S6 — EPISTEMIC BOUNDARIES AND EXCLUDED INTERPRETATIONS

S6.1 Demarcating EFT from Non-Falsifiable Frameworks

EFT is formulated as a falsifiable scientific framework whose principal predictions are operationally defined in the main manuscript. It does not propose to explain phenomenal consciousness in its entirety and explicitly acknowledges the hard problem as an unresolved philosophical challenge that EFT's framework does not address.

S6.2 Explicit Disavowal of Quantum Consciousness Frameworks

EFT does not endorse, incorporate, or imply the Orchestrated Objective Reduction (Orch-OR) hypothesis (Hameroff & Penrose, 1998). The empirical evidence required to establish sustained, functionally relevant quantum coherence in neural microtubules as a mechanism of conscious integration remains insufficient and contested. EFT therefore makes no quantum-coherence assumption at the level of its mechanistic or formal architecture. More broadly, EFT does not endorse any form of quantum consciousness claim proposing macroscopic quantum computation as a mechanism for conscious integration.

S6.3 Explicit Disavowal of Mystical and Non-Falsifiable Interpretations

EFT's concept of autonomic coherence (γ) and the three temporal regimes should not be interpreted through frameworks invoking non-physical causes, vitalistic principles, or non-falsifiable metaphysical claims. EFT does not propose that HRV is a measure of 'spiritual coherence' or any analogue thereof. The conceptual connections drawn between EFT's temporal regimes and Damasio's proto-self, core self, and autobiographical self are explicitly framed as functional alignments, not ontological identifications.

S6.4 Summary: What This Supplementary Material Does NOT Claim

This supplementary material does not claim that quantum computation occurs in warm neural tissue at timescales relevant to conscious integration, or that any proposed mechanism is immune to thermal decoherence by virtue of geometric or non-local character; that the Orchestrated Objective Reduction (Orch-OR) hypothesis is compatible with or endorsed by EFT; that macroscopic quantum coherence is a mechanism for cortical integration as described in EFT's main text; that twistor geometry is implemented

in biological systems or constitutes a mechanistic component of conscious processing — the geometric heuristic in §S2.2 is retained solely as a conceptual placeholder for possible future formal work on state-space accessibility, not as a physical claim about neural tissue; that autonomic coherence γ is a universal physical operator at the level of quantum fields; that biophoton emission plays a necessary or demonstrated role in conscious processing within EFT; that microtubules are proven computational substrates of conscious experience; that the 'Poised Realm' concept (Kauffman) has been empirically validated as a description of neural tissue; that any speculative mechanism in §S2–S4 has been parameterised within EFT's master equation, or that EFT's empirical predictions require confirmation of any Tier-3 mechanism for their validity; that trauma, clinical depression, or psychotherapeutic outcomes are fully explained by ΔT_bio-slow dynamics; that consciousness has been reduced to any physical or molecular substrate; or that any non-falsifiable, mystical, or metaphysical interpretation of EFT is warranted.

> *DEMARCATION: Additional demarcations apply to §S1.2 (phototransduction analogy) and §S1.3 (ΔTs proposal): The visual pathway and the vagal-interoceptive pathway do not form a single continuous anatomical chain. EFT does not support ontological claims regarding 'internal light' or 'light as the substrate of consciousness.' ΔTs does not constitute a formally validated regime at the same epistemic level as ΔT_i, ΔT_p, or ΔT^f. No mechanism discussed in §S1.2 or §S1.3 has been parameterised within EFT's master equation.*

The main text of EFT stands fully independently of all content presented in this Supplementary Material.

**Supplementary Figure S1. Candidate Functional Forms Relating the Observable Estimator (γ̂) to the Observable Proxy (PCI): M0/M1/M2 Model Comparison**
Three candidate curves are shown relating γ̂ to PCI: M0 (null model, flat line — no systematic relationship beyond noise); M1 (monotonic-saturating — PCI rises with γ̂ to a plateau without decline); M2 (peaked/inverted-U — PCI rises toward a maximum near γc, then declines at supracritical γ̂; Aston-Jones & Cohen, 2005; Sara & Bouret, 2012; Beggs & Plenz, 2003; Shew & Plenz, 2013; Maschke et al., 2024). Horizontal axis: γ̂ (dimensionless autonomic estimator; RMSSD/RMSSD_basal is the candidate operationalisation currently in use — see Table 1 of the main manuscript — not frozen as a unique or final identity). Vertical axis: PCI (dimensionless, 0–1). γc shading is model-specific: absent in M0; marking the region of maximal slope in M1; marking the candidate transition/peak region in M2 — in no case a single dashed threshold line. γc is itself defined and calibrated on the γ̂ scale (Table 1), not asserted as a validated property of the latent γ. This comparison is defined over the observable pair (γ̂, PCI); it constrains, but does not directly estimate, the corresponding hypothesis about the latent γ–Q relationship, absent a measurement model linking PCI to Q (see main manuscript §5.1, §5.4). Tier-2 designation applies throughout. Directly supports Predictions P1, P4a, and P4b.

DISCLOSURES (SUPPLEMENTARY)

Conflict of Interest Statement: The author declares no competing interests. No commercial or financial relationships exist with any of the theoretical frameworks, concepts, or tools discussed in this supplementary material.

Author Contributions: ALP: Conceptualization, Writing – Original Draft, Writing – Review & Editing. All speculative extensions are clearly attributed to the corresponding author and do not represent any institutional position.

Use of AI Tools (Supplementary): Generative AI language models — Claude (Anthropic), ChatGPT (OpenAI), and Gemini (Google) — were used for formatting, grammar checking, and DOI verification of citations against Crossref/PubMed/publisher records. All theoretical content, speculative extensions, demarcations, and epistemic labelling are the sole responsibility of the author.

SUPPLEMENTARY REFERENCES

Arnsten, A. F. T. (2015). Stress weakens prefrontal networks: Molecular insults to higher cognition. *Nature Neuroscience*, *18*(10), 1376–1385. https://doi.org/10.1038/nn.4087

Aston-Jones, G., & Cohen, J. D. (2005). An integrative theory of locus coeruleus–norepinephrine function: Adaptive gain and optimal performance. *Annual Review of Neuroscience*, *28*, 403–450. https://doi.org/10.1146/annurev.neuro.28.061604.135709

Attwell, D., & Laughlin, S. B. (2001). An energy budget for signaling in the grey matter of the brain. *Journal of Cerebral Blood Flow & Metabolism*, *21*(10), 1133–1145. https://doi.org/10.1097/00004647-200110000-00001

Barrett, L. F., & Simmons, W. K. (2015). Interoceptive predictions in the brain. *Nature Reviews Neuroscience*, *16*(7), 419–429. https://doi.org/10.1038/nrn3950

Baylor, D. A., Lamb, T. D., & Yau, K. W. (1979). Responses of retinal rods to single photons. *Journal of Physiology*, *288*, 613–634. https://doi.org/10.1113/jphysiol.1979.sp012594

Beggs, J. M., & Plenz, D. (2003). Neuronal avalanches in neocortical circuits. *Journal of Neuroscience*, *23*(35), 11167–11177. https://doi.org/10.1523/JNEUROSCI.23-35-11167.2003

Buzsáki, G., & Draguhn, A. (2004). Neuronal oscillations in cortical networks. *Science*, *304*(5679), 1926–1929. https://doi.org/10.1126/science.1099745

Buzsáki, G. (2006). *Rhythms of the Brain*. Oxford University Press. ISBN: 9780195301069

Connors, B. W., & Long, M. A. (2004). Electrical synapses in the mammalian brain. *Annual Review of Neuroscience*, *27*, 393–418. https://doi.org/10.1146/annurev.neuro.26.041002.131128

Craddock, T. J. A., Friesen, D., Mane, J., Hameroff, S., & Tuszynski, J. A. (2014). The feasibility of coherent energy transfer in microtubules. *Journal of the Royal Society Interface*, *11*(100), 20140677. https://doi.org/10.1098/rsif.2014.0677

Crick, F., & Koch, C. (1990). Toward a neurobiological theory of consciousness. *Seminars in the Neurosciences*, *2*, 263–275. [No DOI — pre-systematic assignment; citation verified via PubMed and Google Scholar]

Damasio, A. R. (1999). *The feeling of what happens: Body and emotion in the making of consciousness*. Harcourt.

Damasio, A. R. (2010). *Self comes to mind: Constructing the conscious brain*. Pantheon Books.

Feldman, H., & Friston, K. J. (2010). Attention, uncertainty, and free-energy. *Frontiers in Human Neuroscience*, *4*, 215. https://doi.org/10.3389/fnhum.2010.00215

Friston, K. (2010). The free-energy principle: A unified brain theory? *Nature Reviews Neuroscience*, *11*(2), 127–138. https://doi.org/10.1038/nrn2787

Friston, K., FitzGerald, T., Rigoli, F., Schwartenbeck, P., & Pezzulo, G. (2017). Active inference: A process theory. *Neural Computation*, *29*(1), 1–49. https://doi.org/10.1162/NECO_a_00912

Friston, K. J., Wiese, W., & Hobson, J. A. (2020). Sentience and the origins of consciousness: From Cartesian duality to Markovian monism. *Entropy*, *22*(5), 516. https://doi.org/10.3390/e22050516

Hameroff, S., & Penrose, R. (1998). Quantum computation in brain microtubules? The Penrose–Hameroff 'Orch OR' model of consciousness. *Philosophical Transactions of the Royal Society A*, *356*(1743), 1869–1896. https://doi.org/10.1098/rsta.1998.0254

Heimburg, T., & Jackson, A. D. (2005). On soliton propagation in biomembranes and nerves. *PNAS*, *102*(28), 9790–9795. https://doi.org/10.1073/pnas.0503823102

Kauffman, S. A. (2016). *Humanity in a creative universe*. Oxford University Press.

Kauffman, S. A. (2019). *A world beyond physics: The emergence and evolution of life*. Oxford University Press.

Kawai, Y. (2018). Differential ascending projections from the male rat caudal nucleus of the tractus solitarius: An interface between local microcircuits and global macrocircuits. *Frontiers in Neuroanatomy*, *12*, 63. https://doi.org/10.3389/fnana.2018.00063

Kukushkin, N. V., & Carew, T. J. (2017). Memory takes time. *Neuron*, *95*(2), 259–279. https://doi.org/10.1016/j.neuron.2017.05.029

Leitner, D. M. (2008). Energy flow in proteins. *Annual Review of Physical Chemistry*, *59*, 233–259. https://doi.org/10.1146/annurev.physchem.59.032607.093606

Leitner, D. M., Pandey, H. D., & Reid, K. M. (2019). Energy transport across interfaces in biomolecular systems. *Journal of Physical Chemistry B*, *123*(45), 9507–9524. https://doi.org/10.1021/acs.jpcb.9b07086

Levin, M. (2012). Morphogenetic fields in embryogenesis, regeneration, and cancer: Non-local control of complex patterning. *BioSystems*, *109*(3), 243–261. https://doi.org/10.1016/j.biosystems.2012.04.005

Levin, M. (2019). The computational boundary of a 'self': Developmental bioelectricity drives multicellularity and scale-free cognition. *Frontiers in Psychology*, *10*, 2688. https://doi.org/10.3389/fpsyg.2019.02688

Levin, M., & Martyniuk, C. J. (2018). The bioelectric code: An ancient computational medium for dynamic control of growth and form. *BioSystems*, *164*, 76–93. https://doi.org/10.1016/j.biosystems.2017.08.011

Martin, K. C., & Kosik, K. S. (2002). Synaptic tagging—who's it? *Nature Reviews Neuroscience*, *3*(10), 813–820. https://doi.org/10.1038/nrn942

Maschke, C., O'Byrne, J., Colombo, M. A., Boly, M., Gosseries, O., Laureys, S., et al. (2024). Critical dynamics in spontaneous EEG predict anesthetic-induced loss of consciousness and perturbational complexity. *Communications Biology*, *7*, 946. https://doi.org/10.1038/s42003-024-06613-8

McEwen, B. S. (2007). Physiology and neurobiology of stress and adaptation: Central role of the brain. *Physiological Reviews*, *87*(3), 873–904. https://doi.org/10.1152/physrev.00041.2006

McEwen, B. S., & Wingfield, J. C. (2003). The concept of allostasis in biology and biomedicine. *Hormones and Behavior*, *43*(1), 2–15. https://doi.org/10.1016/S0018-506X(02)00024-7

Nestler, E. J. (2014). Epigenetic mechanisms of drug addiction. *Neuropharmacology*, *76*, 259–268. https://doi.org/10.1016/j.neuropharm.2013.04.004

Northoff, G., & Huang, Z. (2017). How do the brain's time and space mediate consciousness and its different dimensions? Temporo-spatial theory of consciousness (TTC). *Neuroscience & Biobehavioral Reviews*, *80*, 630–645. https://doi.org/10.1016/j.neubiorev.2017.07.013

Penrose, R. (1967). Twistor algebra. *Journal of Mathematical Physics*, *8*(2), 345–366. https://doi.org/10.1063/1.1705200

Sara, S. J., & Bouret, S. (2012). Orienting and reorienting: The locus coeruleus mediates cognition through arousal. *Neuron*, *76*(1), 130–141. https://doi.org/10.1016/j.neuron.2012.09.011

Schoenlein, R. W., Peteanu, L. A., Mathies, R. A., & Shank, C. V. (1991). The first step in vision: Femtosecond isomerization of rhodopsin. *Science*, *254*(5030), 412–415. https://doi.org/10.1126/science.1925597

Shew, W. L., & Plenz, D. (2013). The functional benefits of criticality in the cortex. *The Neuroscientist*, *19*(1), 88–100. https://doi.org/10.1177/1073858412445487

Sherman, S. M., & Guillery, R. W. (2002). The role of the thalamus in the flow of information to the cortex. *Philosophical Transactions of the Royal Society B*, *357*(1428), 1695–1708. https://doi.org/10.1098/rstb.2002.1161

Si, K., Lindquist, S., & Kandel, E. R. (2003). A neuronal isoform of the Aplysia CPEB has prion-like properties. *Cell*, *115*(7), 879–891. https://doi.org/10.1016/s0092-8674(03)01020-1

Singer, W., & Gray, C. M. (1995). Visual feature integration and the temporal correlation hypothesis. *Annual Review of Neuroscience*, *18*, 555–586. https://doi.org/10.1146/annurev.ne.18.030195.003011

Tang, R., & Dai, J. (2014). Biophoton signal transmission and processing in the brain. *Journal of Photochemistry and Photobiology B*, *139*, 71–75. https://doi.org/10.1016/j.jphotobiol.2013.12.008

Tuszynski, J. A., Hameroff, S., Sataric, M. V., Trpisova, B., & Nip, M. L. A. (1995). Ferroelectric behavior in microtubule dipole lattices: Implications for information processing, signalling and assembly/disassembly. *Journal of Theoretical Biology*, *174*(3), 371–380. https://doi.org/10.1006/jtbi.1995.0106

Yu, A. J., & Dayan, P. (2005). Uncertainty, neuromodulation, and attention. *Neuron*, *46*(4), 681–692. https://doi.org/10.1016/j.neuron.2005.04.026
